# Clonal CD8 T Cells Accumulate in the Leptomeninges and Communicate with Microglia in Human Neurodegeneration

**DOI:** 10.21203/rs.3.rs-3755733/v1

**Published:** 2024-01-24

**Authors:** Ryan Hobson, Samuel H.S. Levy, Delaney Flaherty, Harrison Xiao, Benjamin Ciener, Hasini Reddy, Chitra Singal, Christine Y. Kim, Andrew F. Teich, Neil A. Shneider, Elizabeth M. Bradshaw, Wassim Elyaman

**Affiliations:** 1 Division of Translational Neurobiology, Columbia University Irving Medical Center, New York, NY, 10032, USA.; 2 Department of Neurology, Columbia University Irving Medical Center, New York, NY, 10032, USA.; 3 Department of Pathology and Cell Biology, Columbia University Irving Medical Center, New York, NY 10032.; 4 The Taub Institute for Research on Alzheimer’s Disease and the Aging Brain, Columbia University Irving Medical Center, New York, NY, 10032, USA.; 5 The Eleanor and Lou Gehrig ALS Center, Columbia University Irving Medical Center, New York, 10032, USA.; 6 The Center for Motor Neuron Biology and Disease, Columbia University Irving Medical Center, New York, 10032, USA.; 7 The Carol and Gene Ludwig Center for Research on Neurodegeneration, Columbia University Irving Medical Center, New York, NY, 10032, USA.

## Abstract

Murine studies have highlighted a crucial role for immune cells in the meninges in surveilling the central nervous system (CNS) and influencing neuroinflammation. However, how meningeal immunity is altered in human neurodegeneration and its effects on CNS inflammation is understudied. We performed the first single-cell analysis of the transcriptomes and T cell receptor (TCR) repertoire of 104,635 immune cells from 55 postmortem human brain and leptomeningeal tissues from donors with neurodegenerative diseases including amyotrophic lateral sclerosis, Alzheimer’s disease, and Parkinson’s disease. RNA and TCR sequencing from paired leptomeninges and brain allowed us to perform lineage tracing to identify the spatial trajectory of clonal T cells in the CNS and its borders. We propose that T cells activated in the brain emigrate to and establish residency in the leptomeninges where they likely contribute to impairments in lymphatic drainage and remotely to CNS inflammation by producing IFNγ and other cytokines. We identified regulatory networks local to the meninges including NK cell-mediated CD8 T cell killing which likely help to control meningeal inflammation. Collectively, these findings provide not only a foundation for future studies into brain border immune surveillance but also highlight important intercellular dynamics that could be leveraged to modulate neuroinflammation.

## Introduction

The meninges are a supportive scaffold that maintains the structural integrity of the central nervous system (CNS) and are composed of three main layers: the dura, arachnoid, and pia. In mice, meningeal lymphatics in the dura drain soluble antigens released into the cerebrospinal fluid (CSF) through the brain’s glymphatic system [1]. In mice, the meninges harbor a unique resident macrophage population that directly interacts with and presents antigen to T cells [2, 3], and accumulation and changes in T cell phenotype in aging mouse meninges are linked to impairments in glymphatic flux and exacerbate amyloid pathology and neuroinflammation [4].

Human imaging studies have established impaired glymphatic flow as an important feature of Parkinson’s disease (PD) [5, 6] and increases in leukocyte density and adaptive immune signaling were observed in arachnoid granulations over the course of healthy aging in postmortem human tissue sections [7]. While limited single nucleus RNA and T cell receptor (TCR) sequencing in human meningioma and paired dura [8] as well as in Alzheimer’s disease (AD) leptomeninges [9] have provided insight into the cellular composition of the human meninges, a complex view of the human leptomeninges immune landscape and its potential effects on neuroinflammation, especially in the context of neurodegeneration, are lacking.

Here, we performed single-cell RNA and TCR sequencing on 104,635 immune cells from fresh postmortem leptomeninges and brains acquired through the New York Brain Bank (NYBB) at Columbia University. In addition to serving as a platform for high-dimensional identification of diverse meningeal immune subsets, we found that the meninges harbor substantial numbers of clonally expanded T cells, and samples across several neurodegenerative conditions provide insights into disease-specific alterations in meningeal immunity. We further leverage our dataset to make inferences about the activation and control of T cell activity in the meninges and its relationship to inflammatory changes in the human brain.

## Results

### Leptomeninges Cell Cluster Identification

**Figure 1:**
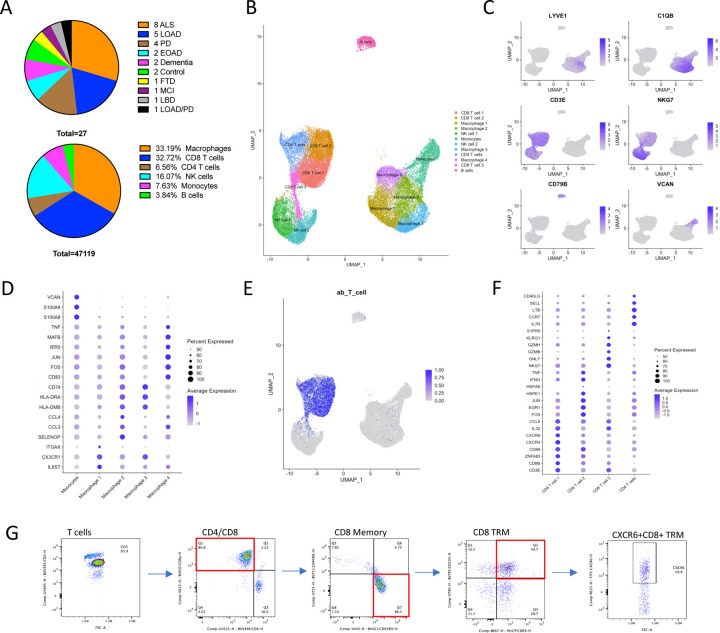
The leptomeninges are a diverse immunological niche. (**A**) Pie charts of the number of samples per disease (top) and the relative proportion of broad cell types in the leptomeninges (bottom). (**B**) UMAP depicting 12 distinct immune cell clusters identified in the human leptomeninges identified by the K-nearest neighbor clustering algorithm. (**C**) Feature plots showing the expression of key marker genes in specific clusters including markers for resident meningeal macrophages (LYVE1, C1QB), T cells (CD3E), NK cells (NKG7), B cells (CD79B), and monocytes (VCAN). (**D**) DotPlot of myeloid sub-cluster defining genes. Rows depict key marker genes, and columns represent distinct clusters. The blue color intensity represents the average expression of the corresponding marker in a cell cluster, while the size of the dot corresponds to the percentage of cells in that cluster that express the gene. (**E**) UMAP depicting alpha/beta TCR positive cells in blue. (**F**) DotPlot of T cell sub-cluster defining genes. Rows depict key marker genes, and columns represent distinct clusters. The blue color intensity represents the average expression of the corresponding marker in a cell cluster, while the size of the dot corresponds to the percentage of cells in that cluster that express the gene. (**G**) Representative flow cytometry plots of tissue resident memory CD8 T cells isolated from the leptomeninges of an ALS patient. Cells were pre-gated for CD45+PI- cells.

The samples collected in this study include 27 postmortem leptomeninges from patients with various neurodegenerative clinical diagnoses summarized in Figure 1A. Relevant demographic, clinical, and histopathological measures are also included in Supplementary File 1. After strict pre-processing (See Methods), we retained transcriptomes from 47,119 meningeal immune cells (Figure 1A). Using Seurat [10] to integrate and cluster the meningeal immune cells, we identified 12 distinct immune clusters (Figure 1B). Canonical markers (Figure 1C) broadly identified major cell types including 4 clusters of meningeal macrophages (C1QB, LYVE1), 4 T cell clusters (CD3E), 2 NK cell clusters (NKG7), a monocyte cluster (VCAN), and a B cell cluster (CD79B). Using the FindAllMarkers function, we further identified marker genes that distinguish each macrophage cluster and identified gene ontology terms that summarize their potential phenotypes (Figure 1D; Supplementary File 2).

T cells expressing the same TCR are derived from the clonally expanded progeny of a parent T cell. With this in mind, we leveraged our paired single cell RNA and TCR sequencing data to infer the developmental relationship and phenotype of each of our T cell clusters. Indeed, all T cell clusters contained cells expressing an alpha/beta TCR (Figure 1E). However, CD8 T cell cluster 3 had very little TCR repertoire overlap with the remaining CD8 T cell clusters (Supplementary Figure 1A), suggesting that they are derived from a separate lineage and, compared to CD8 T cell 1 and 2, expressed higher levels of S1PR5 and ZEB2 (Figure 1F; Supplementary Figure 1B), the simultaneous downregulation of which is necessary for establishing tissue residency [11]. On the other hand, CD8 T cell 1 and 2 expressed high levels of the tissue residency regulator ZNF683 (Hobit) (Supplementary Figure 1B; Figure 1F) and shared significant TCR sequence overlap with each other suggesting that they are developmentally related tissue resident CD8 T cells (Supplementary Figure 1A). Performing differential expression analysis between CD8 T cell 1 and 2 implied an activation trajectory between the two clusters: while CD8 T cell 1 expressed higher levels of chemokine receptors CXCR4 and CXCR6 (Figure 1F; Supplementary Figure 1C), CD8 T cell 2 had a more activated expression profile with high levels of activation-associated transcription factors (FOS, JUN) and pro-inflammatory genes (IFNG, TNF) (Figure 1F; Supplementary Figure 1C). We validated these findings via flow cytometry and confirmed that the majority of T cells in the leptomeninges are tissue resident memory CD8 T cells (CD8+CD45RO+CD45RA-CD69+CD103+) with >50% of the cells expressing CXCR6 (Figure 1G), an important receptor for maintaining CD8 T cell brain residency and survival [12]. Notably, although activated memory CD4 T cells (CD4+CD45RO+CD45RA-CD69+) were also present in the meninges, they mostly did not express CD103 (Supplementary Figure 1D), suggesting that meningeal CD4 T cells are either not tissue resident or that meningeal CD4 T cell residency is maintained by a distinct mechanism.

### Clonally Expanded T Cells Accumulate in the Meninges During Neurodegeneration

Amyloid-beta and alpha-synuclein reactive T cells were found in the blood of patients with late onset AD (LOAD) [13] and PD [14], respectively, and although recent reports show elevated clonal expansion of numerous TCRs in the CSF over the normal course of aging [15] and in patients with mild cognitive impairment (MCI), LOAD, and PD [16], whether T cell clonal expansion occurs in the leptomeninges during neurodegeneration has not been studied. Leveraging our paired RNA and TCR profiling, we found that expanded TCRs (TCRs found in two or more cells) were almost exclusively expressed in CD8 T cells (Supplementary Figure 1E). Examining the top clone in each sample revealed that the leptomeninges are a site of striking T cell clonal expansion, with 3–40% of a given patient’s overall meningeal clonal repertoire being represented by a single TCR (Supplementary Figure 1F).

**Figure 2:**
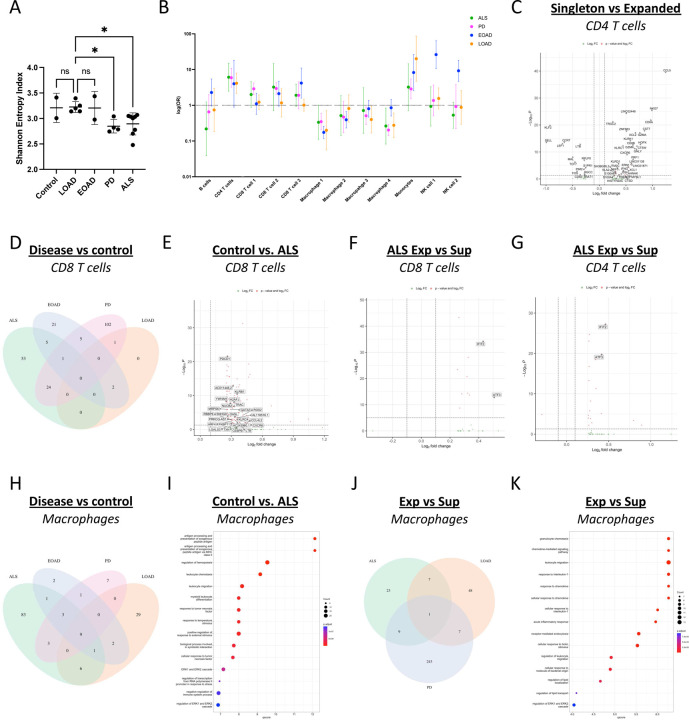
Expanded T cells in the neurodegenerative leptomeninges interact with border macrophages. (**A**) Shannon entropy measurement of TCR clonal diversity across disease conditions. Error bars depict one standard deviation above and below the mean. One-way ANOVA with post-hoc comparison to LOAD. *=p<0.05. (**B**) Odds ratio of statistical difference in each single cell cluster between controls and each neurodegenerative disease using MASC. (**C**) Volcano plot of differentially expressed genes between Expanded (T cells with receptor expressed in 2 or more cells) or singleton meningeal CD4 T cells. (**D**) Venn Diagram of genes commonly upregulated in meningeal CD8 T cells from each neurodegenerative disease in comparison to control. (**E**) Differentially expressed genes upregulated in both ALS and PD CD8 T cells. (**F**) Genes differentially elevated in CD8 T cells from ALS patients with Expanded (negative log2FC) and Superexpanded (positive log2FC). (**G**) Differentially elevated genes in CD4 T cells from ALS patients with Expanded (negative log2FC) and Superexpanded (positive log2FC). (**H**) Venn Diagram of genes commonly upregulated in meningeal macrophages from each neurodegenerative disease in comparison to control. (**I**) GSEA of genes upregulated in ALS in comparison to control meningeal macrophages. (**J**) Venn Diagram of genes commonly upregulated in meningeal macrophages with relatively high levels of clonal expansion from each neurodegenerative disease (**K**) GSEA of genes upregulated in ALS macrophages from patients with Superexpanded meningeal TCR repertoires in comparison to ALS patients with lower levels of meningeal T cell clonal expansion.

To discern the link between meningeal immunity and neurodegeneration, we measured TCR clonal diversity using Shannon entropy. This revealed that amyotrophic lateral sclerosis (ALS) and PD TCR repertoires are less diverse, or more clonally expanded, compared to other conditions (Figure 2A). Although we found no age-related Shannon entropy associations (Supplementary Figure 1G–H), we found that males in our cohort had significantly lower TCR Shannon entropy scores (Supplementary Figure 1I). We then applied Mixed-effects modeling of Associations of Single Cells (MASC) to adjust for demographic effects and associate our single-cell clusters with disease status. Although most clonally expanded T cells detected in our dataset were CD8, CD4 T cells were increased or trended towards increased proportion in all neurodegenerative conditions relative to controls (Figure 2B). Consistent with the Shannon entropy measures, the tissue resident CD8 T cell clusters (CD8 T cell 1 and CD8 T cell 2) were elevated or trending towards increased levels in PD and ALS, respectively (Figure 2B), relative to controls. We also aimed to pinpoint antigenic triggers for T cell clonal expansion by analyzing TCR sequences in highly expanded T cells via Levenshtein distance (Supplementary Figure 2), a language model for assessing the edit distance between character strings. However, our findings revealed no significant homology across patients, possibly due to high HLA haplotype diversity in our cohort.

We investigated the inflammatory phenotype of expanded meningeal T cells, finding that CD8 T cells with hyperexpanded (5% or more of the TCR repertoire) TCRs expressed increased levels ZNF683 and CXCR6 and lower levels of CD27 (Supplementary Figure 3A), indicative of mature tissue residency. Expanded CD4 T cells also displayed higher levels of ZNF683, CXCR6, and activation markers (CCL5, IFNγ, GZMA, GZMK) (Figure 2C) but reduced SELL and CCR7 (Figure 2C), aligning with previous findings on T cell drainage inefficiencies in aged mouse meninges [1, 4].

To determine if neurodegeneration prompts transcriptional changes in meningeal CD8 T cells, we analyzed their profiles across each neurodegenerative disease versus control. Notably, PDCD1 (PD-1) was the most significant upregulated gene in ALS CD8 T cells (Figure 2E). Interestingly, clonally expanded PD-1+CD49d+ CD8 T cells with preferential reactivity towards CNS antigens were increased in the brain, blood, and spinal cord in pre-symptomatic and symptomatic phases in an ALS mouse model [17]. Our analysis also revealed exclusive upregulation of 24 genes (adjusted p-value <0.05) (Figure 2D), including CXCR6 and NK receptors KLRB1 and KLRC4 (Figure 2E; Supplementary Figure 3B), in ALS and PD CD8 T cells, indicating that they may by primed for tissue retention and innate-like effector functions.

Assessing the impact of TCR diversity on meningeal CD8 T cell phenotypes across ALS, LOAD, and PD, we correlated Shannon entropy scores with CD8 T cell gene expression profiles. Although there were many upregulated genes in each disease comparison (Supplementary Figure 3C), only two genes were upregulated in Superexpanded CD8 T cells in both ALS and PD including ATF3 (Figure 2F; Supplementary Figure 3D), a transcriptional modulator of IFNγ [18]. In line with these findings, Gene Ontology revealed an elevation in interferon gamma response factors including IFIT2 in Superexpanded ALS samples (Supplementary Figure 3E). Performing the same analysis on CD4 T cells yielded similar results with elevation of interferon production and response-related pathways and genes (IFIT2, IFITM3, ATF3) in Superexpanded ALS and PD, but not LOAD, CD4 T cells (Figure 2G; Supplementary Figure 3F–G). Altogether, these findings suggest that T cells in the ALS and PD meninges contribute and respond to an IFNγ-rich environment in neurodegeneration.

We next asked whether meningeal macrophages locally influence T cell activation in the meninges. Comparing the gene expression of meningeal macrophages from each neurodegenerative disease against non-neurodegenerative control macrophages revealed conservation of several upregulated genes (Figure 2H) and pathways including MHC class II antigen processing and presentation (HLA-DRB1, HLA-DRA, CD74) (Figure 2I; Supplementary Figure 4A–D). Although it is possible that upregulation of MHC class II is a result of exposure to IFNγ from preactivated T cells arriving in the meninges rather than an initiating antigen presentation event, this nonetheless demonstrates a neurodegeneration-dependent intercellular interaction between meningeal macrophages and T cells. We further asked whether higher levels of meningeal CD8 T cell clonal expansion is related to local changes in resident macrophages. Stratifying ALS, PD, and LOAD samples independently on their levels of clonal expansion revealed an association between clonal expansion and elevated macrophage IL1B transcripts (Figure 2J; Supplementary Figure 4E–G) and IL-1 response gene programs in all disease contexts (Figure 2K; Supplementary Figure 4E–G). IL1B can enhance CD8 T cell proliferation and survival directly through IL1R signaling [19]. Interestingly, the IL1R antagonist IL1RN was also one of the most highly upregulated genes in macrophages from Superexpanded LOAD samples (Supplementary Figure 4G) which may help to restrain IL1B-mediated effects on CD8 T cell proliferation in AD. Collectively, these data suggest that meningeal macrophages not only play a role in local antigen presentation to T cells but may also tune the degree of T cell clonal expansion by modulating IL1B and its antagonists.

### Meningeal T Cells Are Regulated by Local Intercellular Dynamics

**Figure 3:**
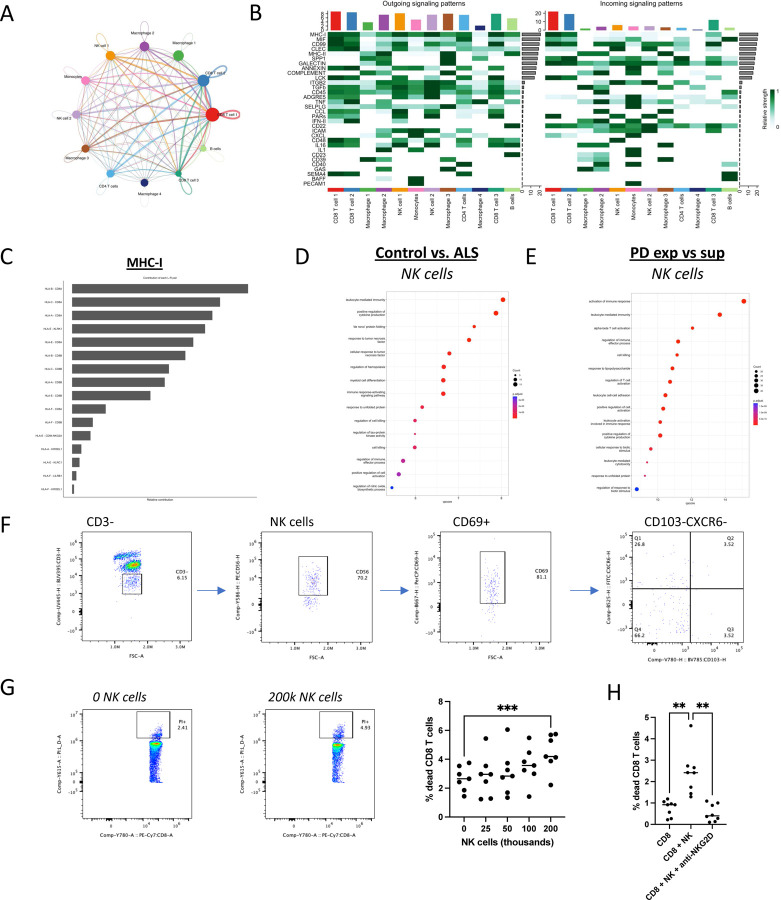
Meningeal T cell clonal expansion is regulated by local intercellular communication networks. (**A**) String diagram of meningeal ligand/receptor interactions inferred using CellChat. Edge width represents the interaction weight/strength. (**B**) Heatmap of detected intercellular interactions identified with CellChat. The intensity of green color represents the importance of a given cluster in contributing to the detected intercellular communication as a Sender and/or Receiver. (**C**) Relative contribution of Ligand/receptor pairs to MHC-I signaling network. (**D**) Gene Ontology terms inferred using genes identified as upregulated in ALS meningeal NK cells in comparison to control. (**E**) Gene Ontology terms inferred using genes identified as upregulated in NK cells from Superexpanded PD samples in comparison to Expanded PD samples. (**F**) Flow cytometric analysis of NK cells in live cells from ALS meninges. Cells were pre-gated on CD45+PI- cells (**G**) Representative flow plots and quantification of percentage of human CD8+ anti-CD3/CD28/CD2 bead stimulated cells that were dead (PI+) after 3 days in culture alone or with increasing amounts of allogeneic NK cells. All conditions n=7. One-way ANOVA with post-hoc comparison. ***p<0.001. (**H**) Quantification of percentage of human CD8+ anti-CD3/CD28/CD2 bead stimulated cells that were dead (PI+) after 4 days in culture alone or with 200k allogeneic NK cells +/- anti-NKG2D neutralizing antibody. All condition n=8. One-way ANOVA with post-hoc comparison. **p<0.01.

Employing the CellChat R package, we inferred key immune interactions within the human meninges. The analysis indicated a robust immune communication network (Figure 3A), particularly involving IFNγ (IFN-II) signaling from CD8 T cells to macrophages (Figure 3B). Other predicted interactions including SPP1 (osteopontin) and GALECTIN (Figure 3B), two important regulators of T cell activation [20] and survival [21], respectively, suggest intrinsic regulation of T cell activity despite active macrophage antigen presentation. As expected, MHC class I signaling emerged as a significant signal potentially output by all cells to varying degrees to CD8 T cells (Figure 3B). Interestingly, we also inferred a strong MHC class I signal between NK cells and CD8 T cells via NK cell receptor complexes (KLRK1, CD94:NKG2A, KLRC1, KIR3DL1) and non-classical MHC class I (HLA-E) (Figure 3C). IFNγ secreted by NK cells licensed in the gut can stimulate astrocyte-mediated killing of T cells in the mouse meninges [22], but whether meningeal NK cells also directly regulate T cells has not been explored. Indeed, NK cells in ALS and PD meninges expressed higher levels of positive and negative regulators of cell killing (Figure 3D; Supplementary Figure 5A–B), while few or no differentially expressed genes were detected in EOAD (Supplementary Figure 5C) and LOAD NK cells, respectively, indicating that NK cell killing activity may be specifically altered in neurodegenerative diseases with higher levels of clonal expansion. Thus, we stratified samples from each disease by clonal expansion and found that NK cell killing genes, especially GZMK, were elevated in Superexpanded ALS and PD meningeal NK cells (Figure 3D–E; Supplementary Figure 5D–E). This was specific to ALS and PD as no meaningful differential expression was observed in Superexpanded LOAD NK cells (Supplementary Figure 5F). Tissue resident NK cells engraft inflamed tissues in the resolution phase of inflammation and directly eliminate T cells to reduce risk of post-infectious autoimmunity [23]. Our single cell RNA data indicated that meningeal NK cells were predominately S1PR5 high (Supplementary Figure 5G) suggesting that they are migratory. Using flow cytometry, we confirmed that meningeal NK cells are activated (CD69+) but not tissue resident (CD103-CXCR6-) (Figure 3F). NK cells are known to regulate CD8 T cells by direct and indirect mechanisms in mice [24, 25], and although NK cells can directly kill PMA/ionomycin or SEB treated human CD8 T cells [26, 27], we wondered if a more physiologic T cell activation could elicit a similar effect. Indeed, we found that human NK cells kill autologous anti-CD3/CD28/CD2 stimulated CD8 T cells in a dose-dependent manner (*p*<0.001) (Figure 3G) and blocking the primary NK cell activating receptor identified from our CellChat analysis, NKG2D (KLRK1), with a monoclonal antibody virtually abolished these effects (*p*<0.001) (Figure 3H). Collectively, these data suggest that NK cells can limit CD8 T cell immunity via direct contact and may regulate the degree of CD8 T cell abundance, and ultimately inflammation, in the human meninges.

### Activated CD8 T Cells Exit the Brain and Establish Residency in the Meninges

**Figure 4:**
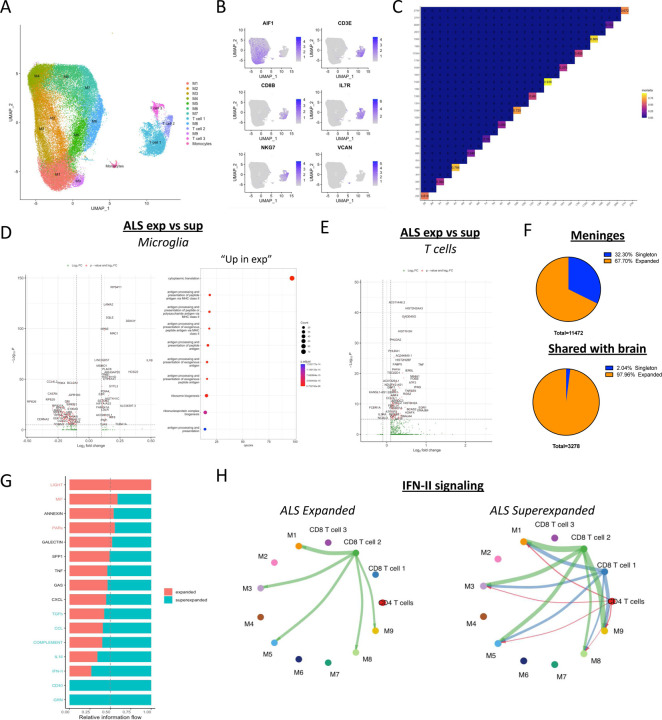
Activated CD8 T cells egress from the brain into the meninges and contribute to neuroinflammation. (**A**) UMAP of cell clusters identified in the brain using the K-nearest neighbor algorithm. (**B**) UMAP highlighting cells expressing key marker genes for microglia (AIF1), Cytotoxic effector memory T cells (CD3E, CD8B, IL7R, NKG7), and monocytes (VCAN). Blue color intensity corresponds to the relative level of expression of the selected gene in a cell. (**C**) Morisita analysis of cross-tissue (brain and meninges) TCR repertoire overlap. (**D**) Volcano plot of differentially expressed genes between microglia in ALS patients with Superexpanded versus Expanded meningeal TCR repertoires (*left*) and GO terms inferred using genes upregulated in microglia from patients with expanded meningeal TCR repertoires (*right*). Positive Log2 fold change corresponds to genes upregulated in Superexpanded microglia, and negative Log2 fold change corresponds to genes downregulated in Superexpanded microglia. (**E**) Volcano plot of differentially expressed genes between brain T cells in ALS patients with Superexpanded versus Expanded meningeal TCR repertoires. (**F**) Pie charts illustrating the percentage of all alpha/beta TCR positive T cells in the meninges that are expanded (top) and all alpha/beta TCR positive T cells in the meninges that also have a TCR that is found in the brain of the same patient that are expanded (bottom). (**G**) Secreted intercellular ligand/receptor interactions inferred between meningeal T cells and microglia from ALS patients using CellChat. Intercellular signaling pattern names highlighted in red on the y-axis denote interactions that were inferred as significantly upregulated in expanded samples, and pattern names highlighted in blue represent interactions that were inferred as significantly upregulated in Superexpanded samples. (**H**) String diagrams depicting differential intercellular IFN-II signaling between meningeal T cells and microglia between Expanded and Superexpanded ALS sample sets. Edge width represents the relative interaction strength detected between two cell clusters.

Meningeal immunity can influence CNS inflammation in mouse models of amyloid-beta aggregation [4, 28]. In tandem, microglia recruit and present antigen to T cells which are required for the development of neurodegenerative pathology in a mouse model of tauopathy [29]. However, whether brain-infiltrating T cells are recruited from the meninges is unclear. To assess the relationship of leptomeningeal immune changes to human brain immunity, we sequenced immune cells from 28 fresh postmortem brain samples across 22 patients, including 14 patients overlapping with our leptomeninges sequencing cohort (Supplementary File 1) using a mechanical dissociation pipeline (See Methods). After strict preprocessing and integration (See Methods), we retained 57,516 brain immune cell transcriptomes and TCRs. In addition to 9 microglia clusters, we identified 3 populations of memory CD8 T cells and one population of monocytes (Figure 4A–B). We first wondered whether T cells travel between the meninges and the brain and reasoned that TCRs shared between the brain and meninges must be derived from the same parent cell and can be used to trace the spatial trajectory of T cells. Indeed, TCRs in the two compartments overlapped, though to a variable extent (Figure 4C). Interestingly, TCR repertoires from ALS meninges exhibited significantly higher TCR repertoire overlap with the corresponding brain TCR repertoire than did LOAD meninges (Supplementary Figure 6A). Given that our ALS and LOAD brain samples came from the motor cortex and the hippocampus, respectively, it is possible that the anatomical distance between the hippocampus and cortical meninges sampled in our cohort explains this disparity in TCR overlap. However, comparing the TCR overlap of meninges and parietal lobe from 3 non-ALS patients showed similarly low repertoire overlap (Supplementary Figure 6B), suggesting that T cell trafficking between the ALS cortex and the meninges is likely specific to ALS. Interestingly, we also found that the TCR sequence overlap between the PD motor cortex and the cortical meninges was higher than that between the substantia nigra and the meninges (Supplementary Figure 6B) which may indicate that meningeal T cell repertoire changes specifically affect the TCR repertoire of the motor cortex.

Given that meningeal T cells appear to enter the brain, we next wondered if meningeal T cells respond to or influence CNS inflammation. First, we found that MHC class II genes (HLA-DRA, HLA-DRB1, CD74) are upregulated in both LOAD (Supplementary Figure 7A) and ALS (Supplementary Figure 7B–C) microglia. Notably, unlike in the meninges, HLA class I genes (HLA-A, HLA-B, HLA-C) were also upregulated in both LOAD and ALS microglia, with slightly stronger upregulation in ALS. This led us to consider that CD8 T cells may be activated in the brain before draining into the meninges. To address this possibility, we compared microglial gene expression between patients with Superexpanded and Expanded meningeal TCR repertoires. Surprisingly, MHC class I genes were lower in microglia from ALS patients with Superexpanded meningeal TCR repertoires (Figure 4D) and higher in microglia from LOAD patients with Superexpanded meningeal TCR repertoires (Supplementary Figure 7D). Notably, several leukocyte migration-related cytokines including CCL4 and CXCL8 were also upregulated in LOAD microglia from patients with Superexpanded meningeal TCR repertoires (Supplementary Figure 7D) whereas IL1B was dramatically increased in ALS microglia from patients with Superexpanded meningeal TCR repertoires (Figure 4D). Next, we assessed the effects of these microglial changes on brain T cells. As expected, brain CD8 T cells expressed higher levels of T cell activation genes in both ALS and LOAD in comparison to control (Supplementary Figure 8A–B). Indeed brain CD8 T cells from ALS patients with Superexpanded meningeal TCR repertoires expressed higher levels of effector cytokines including IFNγ and TNFα (Figure 4E) as well as T cell activation-related gene sets (Supplementary Figure 8C) despite lower levels of microglial HLA-A,B,C. This likely indicates that microglial MHC class I antigen presentation precedes the accumulation of expanded CD8 T cells in the ALS meninges and that microglial antigen presentation is rapidly downregulated after interacting with T cells. On the contrary brain CD8 T cells in LOAD patients with Superexpanded meningeal TCR repertoires expressed lower levels of T cell activation genes including KLRG1, FASLG, and LTB (Supplementary Figure 8D–E) despite upregulated levels of microglial HLA-A,B,C. This, along with the elevated CCL4 may indicate that microglia recruit T cells from the meninges to the brain and that microglial antigen presentation precedes T cell activation in the LOAD brain. Collectively, these findings suggest that microglia likely recruit CD8 T cells from the meninges via cytokine/chemokine signaling and contribute to their antigen-directed activation.

Given that meningeal CD8 T cell clonal expansion is correlated with MHC class I expression in microglia, we wondered if it was possible that enhanced CD8 T cell abundance in the meninges is due to an accumulation of expanded T cells egressed from the brain. Thus, by identifying meningeal CD8 T cells with TCRs also found in the brain (Supplementary Figure 8F), we sought to analyze the activation status and phenotype of CD8 T cells traveling through the two compartments. Although singletons (TCR found in only 1 cell) represented approximately 32% of all meningeal T cells, almost all (97.96%) TCRs that were shared between the brain and meninges were expanded in the meninges (Figure 4F), indicating that meningeal T cell activation and clonal expansion is a requirement for T cells to travel between the brain and the meninges. Next, we compared the gene expression profile of meningeal CD8 T cells with TCRs also found in the brain to all other meningeal CD8 T cells and found that meningeal brain-derived T cells express higher levels of the tissue residency marker ZNF683 [30] and lower levels of CD27 (Supplementary Figure 8G), indicating a more mature phenotype. These findings strongly suggest that CD8 T cells accumulate in the meninges and establish residency after activation in the brain.

Cytokine production by meningeal T cells can have a profound influence on CNS inflammation and behavior in mice [31–33]. Therefore, we next used CellChat to identify potential secreted factors that facilitate communication between meningeal T cells and microglia. By merging and comparing single cell sequencing data from paired brain and meninges samples with low meningeal Shannon entropy and high meningeal Shannon entropy and filtering for potentially secreted signals, we detected 10 putative differentially expressed intercellular communication networks (Figure 4G). In concordance with an elevation in IFNγ and IFN response genes in expanded CD4 and CD8 T cells, respectively, we found IFN-II signaling between meningeal T cells and microglia to be increased in Superexpanded ALS samples (Figure 4H). Indeed, performing the same analysis in LOAD samples yielded similar results (Supplementary Figure 9A–B). In addition to its effects on microglia, IFNγ also impairs lymphatic drainage by dysregulating expression and distribution of claudins on lymphatic endothelial cells in mice [34]. Therefore, these data suggest that microglia and activated meningeal CD8 T cells likely interact in a bidirectional chemokine-mediated communication network with the CNS and that chemokine signaling may be a potential therapeutic target for modulating meningeal T cell-mediated inflammation in the degenerating brain.

## Discussion

In this study, we detailed the immune profiles within human leptomeninges and brains affected by various neurodegenerative diseases, revealing similarities with mouse immune dynamics. We noted that meningeal macrophages likely participate in antigen presentation, impacting CD8 T cell surveillance in the CNS, and identified widespread expression of memory T cell markers on meningeal T cells, suggesting their readiness to combat CNS threats. Clonally expanded memory CD8 T cells were present across all examined conditions, indicating a baseline presence ready for a rapid targeted response. However, the specific triggers for T cell activation remain to be explored in future targeted studies.

Using a combination of computational and experimental approaches, we further highlight local meningeal regulatory mechanisms that likely restrain T cell clonal expansion including direct T cell killing by meningeal NK cells. In addition to identifying dynamic changes in NK cell cytotoxicity gene programs that correlate with disease status and the degree of CD8 T cell clonality, we confirmed that NK cells can directly kill activated CD8 T cells through NKG2D and is one mechanism by which meningeal immunity may counteract excessive neuroinflammation. Although it is unclear if NK cells target all CD8 T cells or a specific subset, these findings could lead to cell therapies designed to modulate CD8 T cell-driven neuroinflammation.

In addition to insights into the dynamic changes that occur locally in the meninges, paired brain and meninges RNA/TCR sequencing from 14 patients allowed us to make important observations about the relationship between meningeal T cell accumulation and CNS immunity. Indeed, TCR sequences were shared among expanded T cells in both the brain and meninges across all samples. Comparing T cell phenotype across diseases and meningeal clonal expansion status also revealed that the increased clonal expansion of CD8 T cells in the meninges is correlated with microglial MHC class I antigen presentation and CD8 T cell activation in the brain. Therefore, it is likely that the appearance of clonally expanded CD8 T cells in the meninges occurs in the aftermath of T cell activity in the brain and is reflective of an accumulation of T cells exiting the brain rather than ongoing local proliferation. Interestingly, knocking out CCR7 in both wild type and 5xFAD mice leads to an accumulation of CD103+ resident T cells in the meninges [4] raising the possibility that in the absence of a robust elimination mechanism, either via elimination by other cells in the meninges (i.e. NK cells) or lymphatic drainage, T cells accumulate and establish residency in the meninges.

Finally, using CellChat we found that accumulated meningeal T cells likely influence microglial inflammation through the production of cytokines including IFNγ. Previous work has shown that meningeal T cell-derived cytokines including IFNγ [33], IL-4 [35], and IL-17a [32] regulate mouse behavior through direct neuronal and microglia-mediated mechanisms. Further, T cell-derived IFNγ impairs mouse dural lymphatic drainage by altering cadherin distribution on lymphatic endothelial cells [34]. It is therefore likely that an accumulation of T cells in the meninges plays an important role in not only indirectly influencing the CNS but also impairing efficient egress of T cells out of the meninges and into draining lymph nodes.

Collectively, our findings implicate the meninges as an important site of T cell activity that may be controlled by both microglial and local meningeal immune cell intercellular dynamics (Supplementary Figure 9C). We identified numerous disease-specific changes indicating impairments in elimination of IFNγ-producing T cells from the meninges which may further exacerbate impairments in lymphatic drainage and directly contribute to neuroinflammation. Numerous recent studies have observed changes in the proteome and immune cell compositions of the CSF in both aging and neurodegenerative contexts [15, 16, 36], paving the way for new potential biomarkers for neurodegenerative disease progression. However, it is unclear how these peripheral changes relate to meningeal, and ultimately CNS immunity. This considered, given its physiologic placement at the border between the periphery and the CNS, studying the intercellular and chemokine-mediated dynamics in the leptomeninges could prove to be an important avenue for developing novel biomarkers and designing drugs to modulate CNS inflammation in certain neurodegenerative contexts.

## Methods

### Source of human brain and leptomeninges

Human postmortem brain and leptomeninges were collected by the New York Brain Bank at Columbia University using an established tissue banking pipeline [37]. After careful dissection of regions of interest, tissues were stored in Hibernate-A medium (Gibco cat. #A1237501) containing B27 serum-free supplement (Gibco cat #17504044) and stored at 4C until ready for further processing.

### Tissue dissociation and sorting

Postmortem tissue was processed immediately after collection and passed through a variety of dissociation and debris removal procedures. Leptomeninges were placed in 1mg/mL of Collagenase D (Roche cat. #51657128) dissolved in PBS and incubated at 37C for 10 minutes. Tissue was then mashed through a 70um strainer using the plunger of a 3mL syringe to achieve a single cell suspension. Cells were subsequently spun at 300g for 10 minutes and subjected to debris removal using a commercially available density gradient-based Debris Removal Solution (Miltenyi cat. #130-109-398). Postmortem brain was mechanically dissociated using a combination of mincing with a razor blade and dounce homogenization in a 15mL glass tissue homogenizer. Dissociated tissues were passed through a 70um filter and cleared of myelin using Myelin Removal Beads (Miltenyi cat. #130-096-433) and the MultiMacs Cell24 Separator. After debris removal, leptomeninges and brain cells were pelleted at 300xg for 10 minutes and incubated with anti-CD11b APC/Cy7 (BioLegend cat. #101226) and anti-CD45 BV510 (BD Biosciences cat. #563204) antibodies at 1:100 dilution in staining buffer (2% FBS in PBS) for 20 minutes. Cells were pelleted and resuspended in staining buffer containing propidium iodide (PI) at a 1:1000 dilution (Sigma Aldrich Cat. #P4864). CD45^+^/PI^-^ cells were sorted on the Sony MA900 (Sony Biotechnology, USA) into the A1 well of a 96 well plate (Eppendorff cat. #951020401) pre-coated with 2% FBS. All sorting was performed using a 100um nozzle.

### 10x Genomics Chromium single cell 5’ library construction

Cell capture, amplification, and library construction was performed on the 10x Chromium platform using the manufacturer’s publicly available protocol. Briefly, after sorting a fraction of cell suspension was used to estimate total cell count and viability. All remaining sorted cells were loaded in the Chromium platform using the 10x Genomics Next GEM Single Cell 5’ v2 for scRNA and TCR-seq. Libraries were prepared using 10x library reagents in accordance with their 5’ RNA and TCR sequencing library protocol and sequenced on the NovaSeq 6000. RNA and TCR reads were aligned to the Hg38 genome and clonotype/contig matrices were generated using Cell Ranger (v6.1.2).

### Pre-processing of paired scRNA-seq and scTCR-seq data

Sequencing data analyses were conducted using R (v4.0.0 for data filtering and sample integration, v4.2.2 for downstream analyses). Barcoded matrices from CellRanger were processed in Seurat (v4.3.0) by retaining features expressed in at least 3 cells. Low quality and dying cells were removed by filtering out cells with fewer than 200 features and greater than 10% of reads aligning to mitochondrial genes, respectively.

### Sample integration and cell cluster annotation

Leptomeninges samples were integrated first by identifying anchor features using Canonical Correlation Analysis (CCA) with the FindIntegrationAnchors function in Seurat. Clustering was then performed using the K-nearest neighbor algorithm utilizing 30 dimensions and a resolution of 0.6. Cluster identities were defined using both accepted canonical markers and genes identified using the FindAllMarkers function. 3 clusters were omitted based on poorly defined markers, existence in few samples, and evidence of mixed cellular identity (myeloid/lymphoid). Gene Ontology was performed using the clusterProfiler (v4.8.2) R package by selecting all positive or negative differentially expressed genes. Brain single cell data were integrated as above and clustered using 30 dimensions and a resolution of 0.6. 3 small clusters were omitted based on poorly defined markers. Meningeal cluster proportions were compared across conditions using Mixed-effects modeling of Associations of Single Cells (MASC) [38] by setting disease as the contrasting feature, batch as a random effect, and sex and age as fixed effect variables.

### TCR sequencing analysis

TCR sequences were analyzed and integrated into the single cell RNA Seurat object using the R package scRepertoire (v1.10.1) [39]. Only TCR sequences containing both a complete CDR3 alpha and beta chain were considered for downstream clonality analyses. TCR data with more than one CDR3 alpha or beta chain detected in a single cell were excluded. TCR clones were considered identical if both the VDJC gene and CDR3 nucleotide sequences for both the alpha and beta chain were identical. Shannon entropy was measured using the clonalDiversity function in scRepertoire. Sample stratification by expansion level was performed by calculating the mean Shannon entropy across the 27 leptomeninges samples and segregating by samples with indices above the mean (Expanded) and below the mean (Superexpanded) of their respective disease groups. PD samples were stratified around the median because of Shannon entropy skewing. Levenshtein distance measurements were performed using the base R function adist. TCRs were first filtered to include only those expressed in 10 or more cells and barcoded with a unique string tag (“_a”, “_b”, etc.) to identify the sample source in downstream analysis and visualization.

### Inference and analysis of intercellular communication using CellChat

Ligand/receptor interactions were inferred using the CellChat (v1.6.1) [40] package in R using standard settings. Cross-tissue CellChat analysis was performed by first subsetting the microglia from the brain sequencing Seurat object and merging with subsetted T cells from the leptomeninges Seurat object using the merge function. The resulting Seurat object was then split using the subset function to generate separate Seurat objects for Expanded and Superexpanded conditions and passed through the CellChat compare pipeline.

### Flow staining

Fresh postmortem leptomeninges from an ALS case were enzymatically dissociated as described above and stained with anti-CD11b:APC-Cy7 (Biolegend cat. #101226), anti-CD3:BUV395 (BD Biosciences cat. #563546), anti-CD4:BUV496 (BD Biosciences cat. #612936), anti-CD8:BV605 (Biolegend cat. #301040), anti-CD45RO:BV421 (Biolegend cat. #304224), anti-CD45RA:BV711 (Biolegend cat. #304138), anti-CD69:PerCP (Biolegend cat. #310927), anti-CD103:BV785 (Biolegend cat. #350229), anti-CXCR6:FITC (Biolegend cat. #356019), and anti-CD56:PE (Biolegend cat. #318306), antibodies in 2% FBS in PBS for 20 minutes. Stained samples were subsequently spun at 300g for 10 minutes and resuspended in 2% FBS containing PI at a dilution of 1:1000. Data were immediately acquired on the Novocyte Penteon.

### NK cell killing assay

CD8 T cells and allogeneic NK cells were isolated from PBMCs from the New York Blood Center (NYBC) using the Miltenyi MACS Human CD8 T cell isolation kit (cat. #130-096-495) and the Miltenyi MACS human NK cell isolation kit (cat. #130-092-657). CD8 T cells were activated with immunoCult Human CD3/CD28/CD2 T Cell Activator beads (cat. #10970) in co-culture with NK cells in the presence of IL-2 (2ng/mL) (R&D Systems cat. #BT-002) and IL-15 (0.1ng/mL) (R&D Systems cat. #BT-015). NKG2D neutralization was performed by first pre-incubating 200k NK cells with 100ug/mL anti-Human CD314 (NKG2D) antibody (Invitrogen cat. #14-5878-82). Cells were spun down at 300xg for 10 minutes after which the antibody concentration was diluted to 50ug/mL for the duration of the 4-day co-culture with anti-CD3/CD28/CD2 activation beads and 100k CD8 T cells in the presence of IL-2 (2ng/mL) and IL-15 (0.1ng/mL). Cell death was assessed by flow cytometry by evaluating the percentage of PI+ cells relative to the number of CD8+ cells (Biolegend cat. #344712).

## Supplementary Material

Supplement 1

## Data Availability

Raw and analyzed sequencing data in this study will be deposited in the NCBI GEO database at the time of publication. Single cell RNA and TCR sequencing analyses will also be made available via an interactive Shiny app.
