# Supplementary material for: Clonal CD8 T Cells Accumulate in the Leptomeninges and Communicate with Microglia in Human Neurodegeneration
Source: Res Sq. 2024 Jan 24:rs.3.rs-3755733. Preprint. [Version 1] doi: 10.21203/rs.3.rs-3755733/v1 (PMC10854309; doi:10.21203/rs.3.rs-3755733/v1)
Supplement: Supplement 1 [file NIHPPrs3755733v1-supplement-1.pdf]

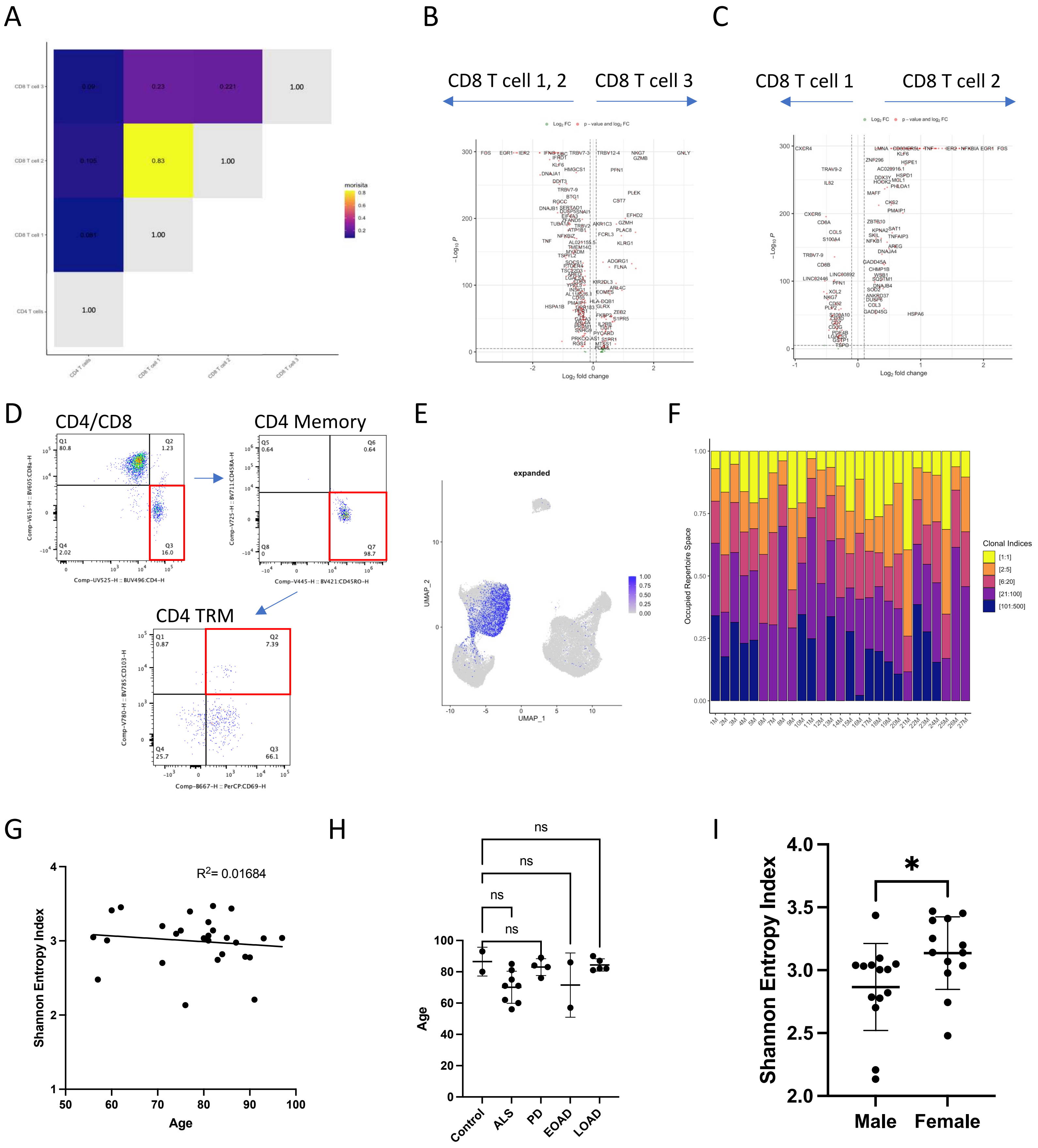
A
B
CD8 T cell 1, 2
CD8 T cell 3
C
CD8 T cell 1
CD8 T cell 2
D
CD4/CD8
CD4 Memory
CD4 TRM
E
expanded
F
Clonal Indices
G
R2= 0.01684
Shannon Entropy Index
Age
H
ns
Age
Control
ALS
PD
EOAD
LOAD
I
Shannon Entropy Index
Male
Female

Supplementary Figure 1: Clonally expanded tissue resident memory CD8 T cells are abundant in the meninges. (**A**) Morisita index measure of TCR repertoire overlap among meningeal T cell clusters. (**B**) Volcano plot of differentially expressed genes between CD8 T cell 3 and all other meningeal CD8 T cells. (**C**) Volcano plot of differentially expressed genes between CD8 T cell 2 and CD8 T cell 1. (**D**) Representative flow cytometry plots of tissue memory CD4 T cells isolated from the leptomeninges of an ALS patient. Cells were pre-gated for CD45+PI- cells. (**E**) UMAP highlighting cells expressing a TCR found in 2 or more cells (expanded). (**F**) Summary of T cell clonal repertoire in each meningeal sample. Colors represent a ranked grouping of TCRs and the height of the rectangle represents the proportion of the sample's TCR repertoire that the group of TCRs occupies. Correlation analysis of meningeal TCR Shannon entropy score against patient sex (**G**) and age (**H**).

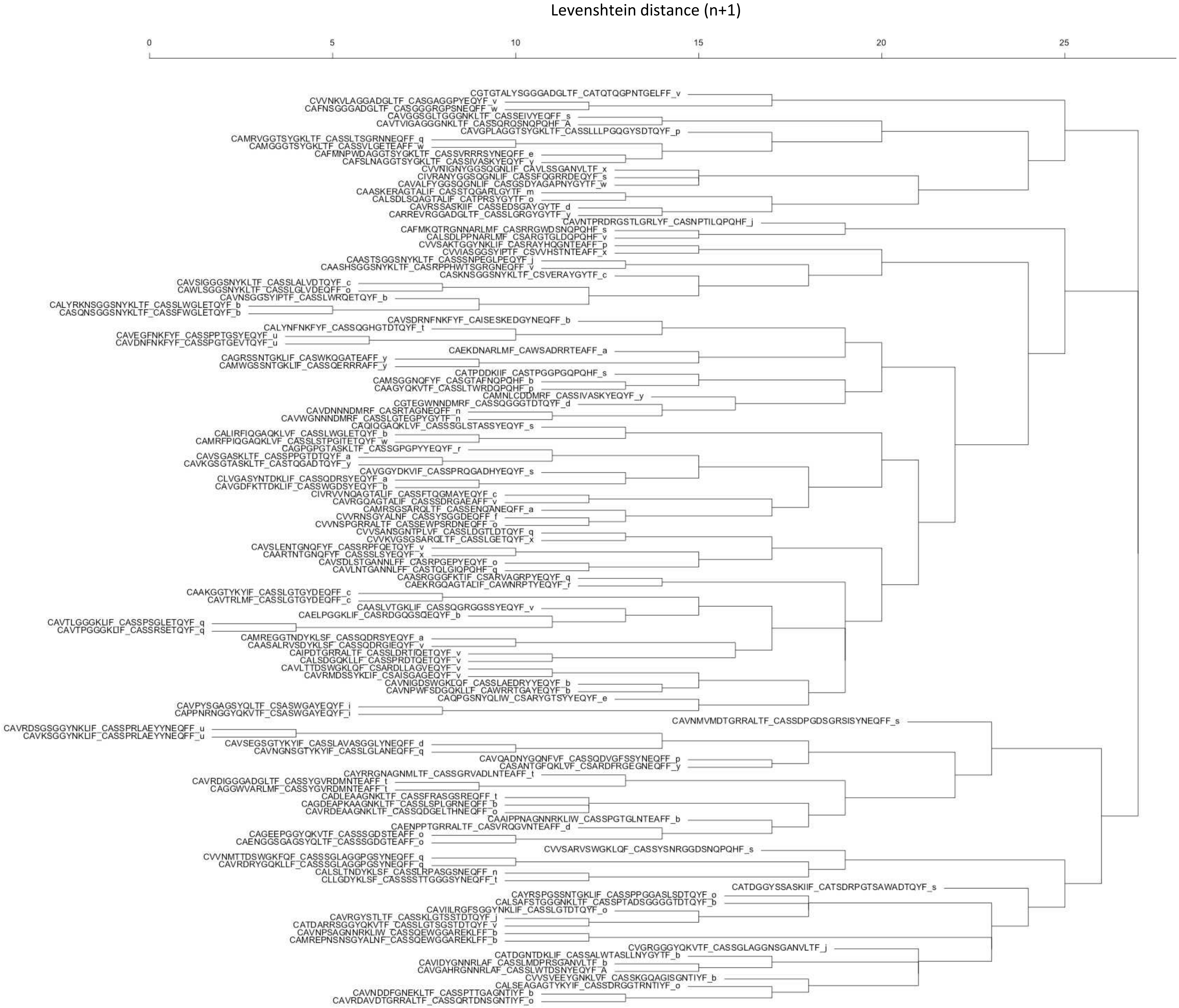

Levenshtein distance (n+1)
0
5
10
15
20
25
CGTGTALYSGGGADGLTF_CATQTQGPNTGELFF_v
CVVNKVLAGGADGLTF_CASGAGGPYEQYF_v
CAFNSGGGADGLTF_CASGGGRGPSNEQFF_w
CAVGGSGLTGGGNKLTF_CASSEIVYEQFF_s
CAVTVIGAGGGNKLTF_CASSQRQSNQPQHF_A
CAVGPLAGGTSYGKLTF_CASSLLLPGQGYSDTQYF_p
CAMRVGGTSYGKLTF_CASSLTSGRNNEQFF_q
CAMGGGTSYGKLTF_CASSVLGETEAFF_w
CAFMNPWDAGGTSYGKLTF_CASSVRRRSYNEQFF_e
CAFSLNAGGTSYGKLTF_CASSIVASKYEQYF_y
CVVNIGNYGGSQGNLIF_CAVLSSGANVLTF_x
CIVRANYGGSQGNLIF_CASSFQGRRDEQYF_s
CAVALFYGGSQGNLIF_CASGSDYAGAPNYGYTF_w
CAASKERAGTALIF_CASSTQGARLGYTF_m
CALSDLSQAGTALIF_CATPRSYGYTF_o
CAVRSSASKIIF_CASSEDSGAYGYTF_d
CARREVRGGADGLTF_CASSLGRGYGYTF_y
CAVNTPRDRGSTLGRLYF_CASNPTILQPQHF_j
CAFMKQTRGNNARLMF_CASRRGWDSNQPQHF_s
CALSDLPPNARLMF_CSARGTGLDQPQHF_v
CVVSAKTGGYNKLIF_CASRAYHQGNTEAFF_p
CVVIASGGSYIPTF_CSVVHSTNTEAFF_x
CAASTSGGSNYKLTF_CASSSNPEGLPEQYF_j
CAASHSGGSNYKLTF_CASRPPHWTSGRGNEQFF_v
CASKNSGGSNYKLTF_CSVERAYGYTF_c
CAVSIGGGSNYKLTF_CASSLALVDTQYF_c
CAWLSGGSNYKLTF_CASSLGLVDEQFF_o
CAVNSGGSYIPTF_CASSLWRQETQYF_b
CALYRKNSGGSNYKLTF_CASSLWGLETQYF_b
CASQNSGGSNYKLTF_CASSFWGLETQYF_b
CAVSDRNFNKFYF_CAISESKEDGYNEQFF_b
CALYNFNKFYF_CASSQGHGTDTQYF_t
CAVEGFNKFYF_CASSPPTGSYEQYF_u
CAVDNFNKFYF_CASSPGTGEVTQYF_u
CAEKDNARLMF_CAWSADRRTEAFF_a
CAGRSSNTGKLIF_CASWKQGATEAFF_y
CAMWGSSNTGKLIF_CASSQERRRAFF_y
CATPDDKIIF_CASTPGGPGQPQHF_s
CAMSGGNQFYF_CASGTAFNQPQHF_b
CAAGYQKVTF_CASSLTWRDQPQHF_p
CAMNLCDDMRF_CASSIVASKYEQYF_y
CGTEGWNNDMRF_CASSQGGGTDTQYF_d
CAVDNNNDMRF_CASRTAGNEQFF_n
CAVWGNNNDMRF_CASSLGTEGPYGYTF_n
CAQIQGAQKLVF_CASSSGLSTASSYEQYF_s
CALIRFIQGAQKLVF_CASSLWGLETQYF_b
CAMRFPIQGAQKLVF_CASSLSTPGITETQYF_w
CAGPGPGTASKLTF_CASSGPGPYYEQYF_r
CAVSGASKLTF_CASSPPGTDTQYF_a
CAVKGSGTASKLTF_CASTQGADTQYF_y
CAVGGYDKVIF_CASSPRQGADHYEQYF_s
CLVGASYNTDKLIF_CASSQDRSYEQYF_a
CAVGDFKTTDKLIF_CASSWGDSYEQYF_b
CIVRVVNQAGTALIF_CASSFTQGMAYEQYF_c
CAVRGQAGTALIF_CASSSDRGAEAFF_v
CAMRSGSARQLTF_CASSENQANEQFF_a
CVVRNSGYALNF_CASSYSGGDEQFF_f
CVVNSPGRRALTF_CASSEWPSRDNEQFF_o
CVVSANSGNTPLVF_CASSLDGTLDTQYF_q
CVVKVGSGSARQLTF_CASSLGETQYF_x
CAVSLENTGNQFYF_CASSRPFQETQYF_v
CAARTNTGNQFYF_CASSSLSYEQYF_x
CAVSDLSTGANNLFF_CASRPGEPYEQYF_o
CAVLNTGANNLFF_CASTQLGIQPQHF_q
CAASRGGGFKTIF_CSARVAGRPYEQYF_q
CAEKRGQAGTALIF_CAWNRPTYEQYF_r
CAAKGGTYKYIF_CASSLGTGYDEQFF_c
CAVTRLMF_CASSLGTGYDEQFF_c
CAASLVTGKLIF_CASSQGRGGSSYEQYF_v
CAELPGGKLIF_CASRDGQGSQEQYF_b
CAVTLGGGKLIF_CASSPSGLETQYF_q
CAVTPGGGKLIF_CASSRSETQYF_q
CAMREGGTNDYKLSF_CASSQDRSYEQYF_a
CAASALRVSDYKLSF_CASSQDRGIEQYF_v
CAIPDTGRRALTF_CASSLDRTIQETQYF_v
CALSDGQKLLF_CASSPRDTQETQYF_v
CAVLTTDSWGKLQF_CSARDLLAGVEQYF_v
CAVRMDSSYKLIF_CSAISGAGEQYF_v
CAVNIGDSWGKLQF_CASSLAEDRYYEQYF_b
CAVNPWFSDGQKLLF_CAWRRTGAYEQYF_b
CAQPGSNYQLIW_CSARYGTSYYEQYF_e
CAVPYSGAGSYQLTF_CSASWGAYEQYF_i
CAPPNRNGGYQKVTF_CSASWGAYEQYF_i
CAVNMVMDTGRRALTF_CASSDPGDSGRSISYNEQFF_s
CAVRDSGSGGYNKLIF_CASSPRLAEYYNEQFF_u
CAVKSGGYNKLIF_CASSPRLAEYYNEQFF_u
CAVSEGSGTYKYIF_CASSLAVASGGLYNEQFF_d
CAVNGNSGTYKYIF_CASSLGLANEQFF_q
CAVQADNYGQNFVF_CASSQDVGFSSYNEQFF_p
CASANTGFQKLVF_CSARDFRGEGNEQFF_y
CAYRRGNAGNMLTF_CASSGRVADLNTEAFF_t
CAVRDIGGGADGLTF_CASSYGVRDMNTEAFF_t
CAGGWVARLMF_CASSYGVRDMNTEAFF_t
CADLEAAGNKLTF_CASSFRASGSREQFF_t
CAGDEAPKAAGNKLTF_CASSLSPLGRNEQFF_b
CAVRDEAAGNKLTF_CASSQDGELTHNEQFF_o
CAAIPPNAGNNRKLIW_CASSPGTGLNTEAFF_b
CAENPPTGRRALTF_CASVRQGVNTEAFF_d
CAGEEPGGYQKVTF_CASSSGDSTEAFF_o
CAENGGSGAGSYQLTF_CASSSGDGTEAFF_o
CVVSARVSWGKLQF_CASSYSNRGGDSNQPQHF_s
CVVNMTTDSWGKFQF_CASSSGLAGGPGSYNEQFF_q
CAVRDRYGQKLLF_CASSSGLAGGPGSYNEQFF_q
CALSLTNDYKLSF_CASSLRPASGSNEQFF_n
CLLGDYKLSF_CASSSSTTGGGSYNEQFF_t
CATDGGYSSASKIIF_CATSDRPGTSAWADTQYF_s
CAYRSPGSSNTGKLIF_CASSPPGGASLSDTQYF_o
CALSAFSTGGGNKLTF_CASSPTADSGGGGTDTQYF_b
CAVIILRGFSGGYNKLIF_CASSLGTDTQYF_o
CAVRGYSTLTF_CASSKLGTSSTDTQYF_j
CATDARRSGGYQKVTF_CASSLGTSGSTDTQYF_v
CAVNPSAGNNRKLIW_CASSQEWGGAREKLFF_b
CAMREPNSNSGYALNF_CASSQEWGGAREKLFF_b
CVGRGGGYQKVTF_CASSGLAGGNSGANVLTF_j
CATDGNTDKLIF_CASSALWTASLLNYGYTF_b
CAVIDYGNNRLAF_CASSLMDPRSGANVLTF_b
CAVGAHRGNNRLAF_CASSLWTDSNYEQYF_A
CVVSVEEYGNKLVF_CASSKGQAGISGNTIYF_b
CALSEAGAGTYKYIF_CASSDRGGTRNTIYF_o
CAVNDDFGNEKLTF_CASSPTTGAGNTIYF_b
CAVRDAVDTGRRALTF_CASSQRTDNSGNTIYF_o

Supplementary Figure 2: Highly expanded meningeal TCRs are not shared between patients. Levenshtein distance measurement among all TCRs expressed in 10 or more cells.

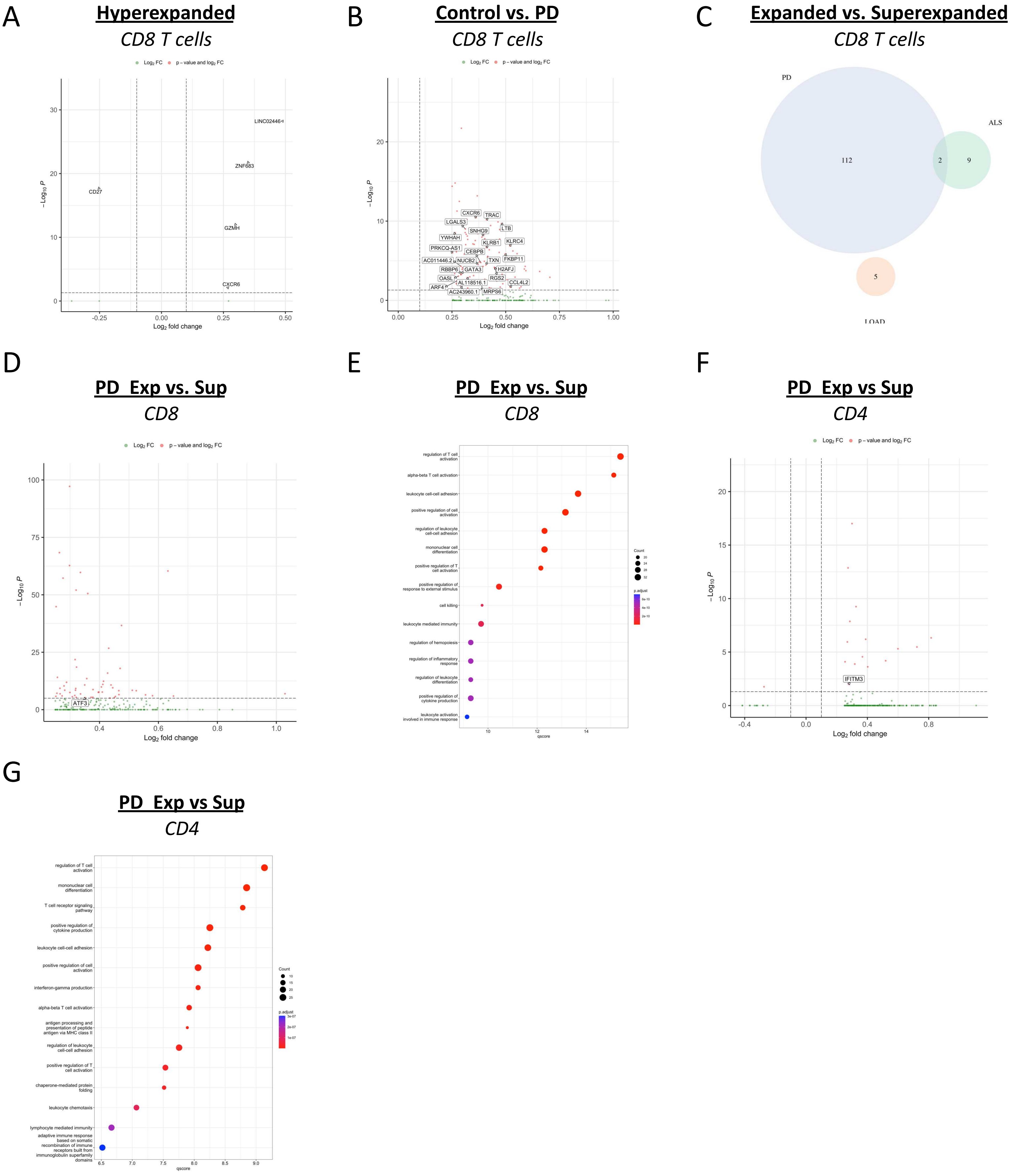
A
Hyperexpanded
CD8 T cells
Log2 FC
p – value and log2 FC
LINC02446
ZNF683
CD27
GZMH
CXCR6
Log2 fold change
−Log10 P
B
Control vs. PD
CD8 T cells
CXCR6
TRAC
LGALS3
SNHG9
LTB
YWHAH
KLRB1
KLRC4
PRKCQ-AS1
CEBPB
AC011446.2
NUCB2
TXN
FKBP11
RBBP6
GATA3
H2AFJ
OASL
RGS2
AL118516.1
CCL4L2
ARF4
AC243960.1
MRPS6
C
Expanded vs. Superexpanded
CD8 T cells
PD
ALS
LOAD
112
2
9
5
D
PD Exp vs. Sup
CD8
ATF3
E
PD Exp vs. Sup
CD8
regulation of T cell activation
alpha-beta T cell activation
leukocyte cell-cell adhesion
positive regulation of cell activation
regulation of leukocyte cell-cell adhesion
mononuclear cell differentiation
positive regulation of T cell activation
positive regulation of response to external stimulus
cell killing
leukocyte mediated immunity
regulation of hemopoiesis
regulation of inflammatory response
regulation of leukocyte differentiation
positive regulation of cytokine production
leukocyte activation involved in immune response
qscore
Count
p.adjust
F
PD Exp vs Sup
CD4
IFITM3
G
PD Exp vs Sup
CD4
regulation of T cell activation
mononuclear cell differentiation
T cell receptor signaling pathway
positive regulation of cytokine production
leukocyte cell-cell adhesion
positive regulation of cell activation
interferon-gamma production
alpha-beta T cell activation
antigen processing and presentation of peptide antigen via MHC class II
regulation of leukocyte cell-cell adhesion
positive regulation of T cell activation
chaperone-mediated protein folding
leukocyte chemotaxis
lymphocyte mediated immunity
adaptive immune response based on somatic recombination of immune receptors built from immunoglobulin superfamily domains
qscore
Count
p.adjust

Supplementary Figure 3: Highly expanded meningeal T cells are IFNγ responsive resident cells. (**A**) Volcano plot of differentially expressed genes between hyperexpanded (T cells with TCR expressed by 5% or more of T cells) versus all other tissue resident CD8 T cells. (**B**) Differentially expressed genes between resident CD8 T cells in PD meninges versus control. (**C**) Overlap between disease groups of genes upregulated in resident CD8 T cells between Superexpanded and Expanded meninges samples. (**D**) Genes differentially expressed between resident CD8 T cells in Superexpanded versus Expanded PD samples. (**E**) Upregulated GO terms inferred from upregulated genes in resident CD8 T cells from patients with Superexpanded meningeal TCR repertoires. (**F**) Genes differentially expressed between resident CD4 T cells in Superexpanded versus Expanded PD samples. (**G**) Upregulated GO terms inferred from upregulated genes in CD4 T cells from patients with Superexpanded meningeal TCR repertoires.

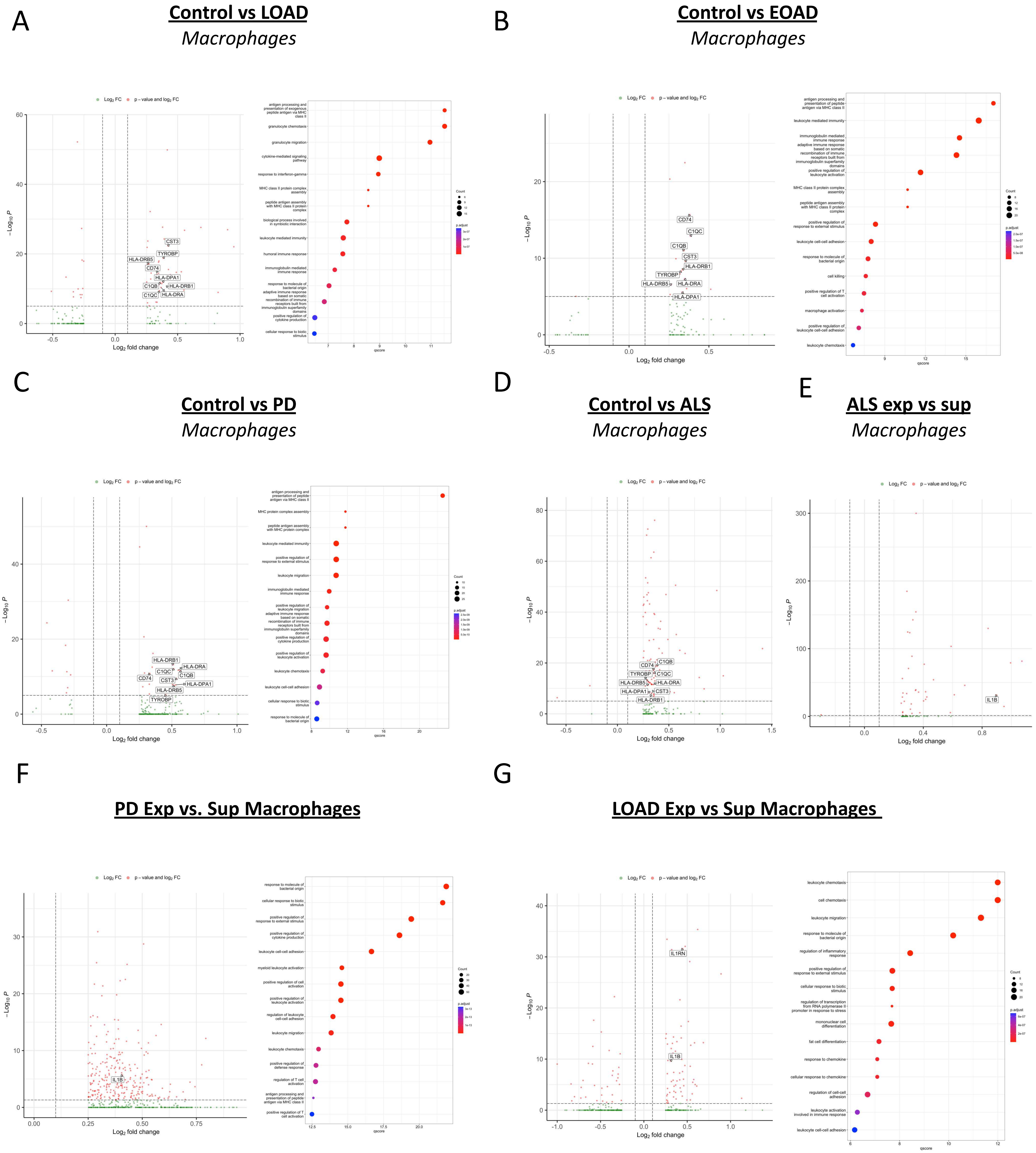

A
Control vs LOAD
Macrophages
Log2 FC
p – value and log2 FC
CST3
TYROBP
HLA-DRB5
CD74
HLA-DPA1
C1QB
HLA-DRB1
C1QC
HLA-DRA
Log2 fold change
−Log10 P
qscore
Count
p.adjust
antigen processing and presentation of exogenous peptide antigen via MHC class II
granulocyte chemotaxis
granulocyte migration
cytokine-mediated signaling pathway
response to interferon-gamma
MHC class II protein complex assembly
peptide antigen assembly with MHC class II protein complex
biological process involved in symbiotic interaction
leukocyte mediated immunity
humoral immune response
immunoglobulin mediated immune response
response to molecule of bacterial origin
adaptive immune response based on somatic recombination of immune receptors built from immunoglobulin superfamily domains
positive regulation of cytokine production
cellular response to biotic stimulus
B
Control vs EOAD
Macrophages
CD74
C1QC
C1QB
CST3
HLA-DRB1
TYROBP
HLA-DRB5
HLA-DRA
HLA-DPA1
antigen processing and presentation of peptide antigen via MHC class II
leukocyte mediated immunity
immunoglobulin mediated immune response
adaptive immune response based on somatic recombination of immune receptors built from immunoglobulin superfamily domains
positive regulation of leukocyte activation
MHC class II protein complex assembly
peptide antigen assembly with MHC class II protein complex
positive regulation of response to external stimulus
leukocyte cell-cell adhesion
response to molecule of bacterial origin
cell killing
positive regulation of T cell activation
macrophage activation
positive regulation of leukocyte cell-cell adhesion
leukocyte chemotaxis
C
Control vs PD
Macrophages
HLA-DRB1
HLA-DRA
C1QC
C1QB
CD74
CST3
HLA-DPA1
HLA-DRB5
TYROBP
antigen processing and presentation of peptide antigen via MHC class II
MHC protein complex assembly
peptide antigen assembly with MHC protein complex
leukocyte mediated immunity
positive regulation of response to external stimulus
leukocyte migration
immunoglobulin mediated immune response
positive regulation of leukocyte migration
positive regulation of cytokine production
positive regulation of leukocyte activation
leukocyte chemotaxis
leukocyte cell-cell adhesion
cellular response to biotic stimulus
response to molecule of bacterial origin
D
Control vs ALS
Macrophages
C1QB
CD74
TYROBP
C1QC
HLA-DRB5
HLA-DRA
HLA-DPA1
CST3
HLA-DRB1
E
ALS exp vs sup
Macrophages
IL1B
F
PD Exp vs. Sup Macrophages
IL1B
response to molecule of bacterial origin
cellular response to biotic stimulus
positive regulation of response to external stimulus
positive regulation of cytokine production
leukocyte cell-cell adhesion
myeloid leukocyte activation
positive regulation of cell activation
positive regulation of leukocyte activation
regulation of leukocyte cell-cell adhesion
leukocyte migration
leukocyte chemotaxis
positive regulation of defense response
regulation of T cell activation
antigen processing and presentation of peptide antigen via MHC class II
positive regulation of T cell activation
G
LOAD Exp vs Sup Macrophages
IL1RN
IL1B
leukocyte chemotaxis
cell chemotaxis
leukocyte migration
response to molecule of bacterial origin
regulation of inflammatory response
positive regulation of response to external stimulus
cellular response to biotic stimulus
regulation of transcription from RNA polymerase II promoter in response to stress
mononuclear cell differentiation
fat cell differentiation
response to chemokine
cellular response to chemokine
regulation of cell-cell adhesion
leukocyte activation involved in immune response
leukocyte cell-cell adhesion

Supplementary Figure 4: Meningeal macrophages contribute to T cell response. (**A**) Differentially expressed genes between LOAD and control meningeal macrophages (*left*) and corresponding upregulated GO terms (*right*). (**B**) Differentially expressed genes between EOAD and control meningeal macrophages (*left*) and corresponding upregulated GO terms (*right*). (**C**) Differentially expressed genes between PD and control meningeal macrophages (*left*) and corresponding upregulated GO terms (*right*). (**D**) Differentially expressed genes between ALS and control meningeal macrophages. (**E**) Differentially expressed genes among meningeal macrophages between ALS samples with Superexpanded versus Expanded TCR repertoires. (**F**) Differentially expressed genes among meningeal macrophages between PD samples with Superexpanded versus Expanded TCR repertoires (*left*) and corresponding upregulated GO terms (*right*). (**G**) Differentially expressed genes among meningeal macrophages between LOAD samples with Superexpanded versus Expanded TCR repertoires (*left*) and corresponding upregulated GO terms (*right*).

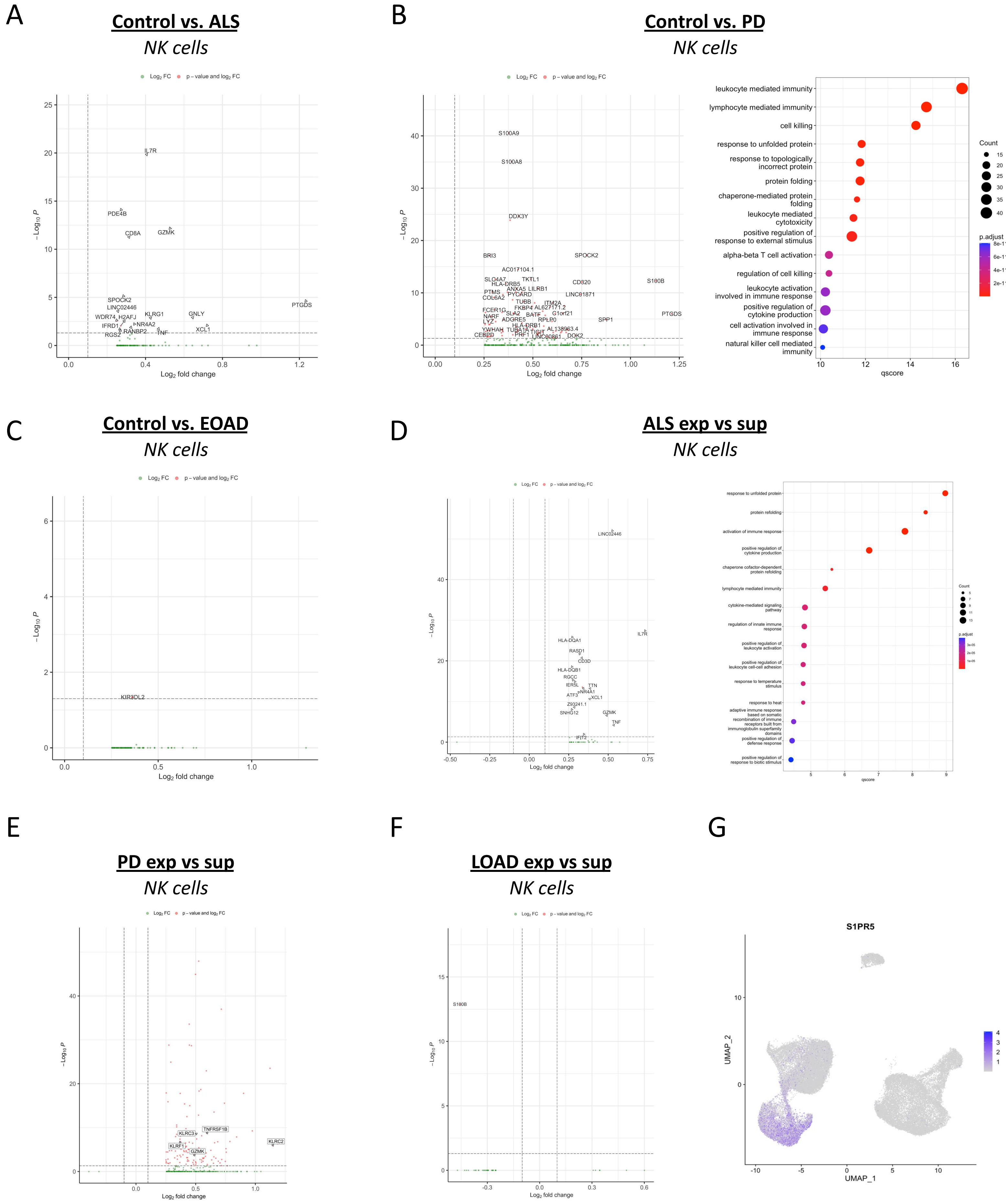
A
Control vs. ALS
NK cells
Log2 FC
p – value and log2 FC
IL7R
PDE4B
CD8A
GZMK
SPOCK2
LINC02446
WDR74
H2AFJ
KLRG1
GNLY
IFRD1
NR4A2
RANBP2
RGS2
TNF
XCL1
PTGDS
Log2 fold change
B
Control vs. PD
NK cells
S100A9
S100A8
DDX3Y
BRI3
SPOCK2
AC017104.1
SLC4A7
TKTL1
HLA-DRB5
CD320
S100B
PTMS
ANXA5
LILRB1
COL6A2
PYCARD
LINC01871
TUBB
ITM2A
FCER1G
FKBP4
AL627171.2
SLA2
BATF
G1orf21
NARF
ADGRE5
RPLP0
PTGDS
LYZ
HLA-DRB1
SPP1
YWHAH
AL138963.4
CEBPD
PRF1
TIGIT
DOK2
leukocyte mediated immunity
lymphocyte mediated immunity
cell killing
response to unfolded protein
response to topologically incorrect protein
protein folding
chaperone-mediated protein folding
leukocyte mediated cytotoxicity
positive regulation of response to external stimulus
alpha-beta T cell activation
regulation of cell killing
leukocyte activation involved in immune response
positive regulation of cytokine production
cell activation involved in immune response
natural killer cell mediated immunity
Count
p.adjust
qscore
C
Control vs. EOAD
NK cells
KIR3DL2
Log2 fold change
D
ALS exp vs sup
NK cells
LINC02446
IL7R
HLA-DQA1
RASD1
CD3D
HLA-DQB1
RGCC
IER5L
TTN
NR4A1
ATF3
XCL1
Z93241.1
SNHG12
GZMK
TNF
IFIT2
Log2 fold change
response to unfolded protein
protein refolding
activation of immune response
positive regulation of cytokine production
chaperone cofactor-dependent protein refolding
lymphocyte mediated immunity
cytokine-mediated signaling pathway
regulation of innate immune response
positive regulation of leukocyte activation
positive regulation of leukocyte cell-cell adhesion
response to temperature stimulus
response to heat
adaptive immune response based on somatic recombination of immune receptors built from immunoglobulin superfamily domains
positive regulation of defense response
positive regulation of response to biotic stimulus
Count
p.adjust
qscore
E
PD exp vs sup
NK cells
TNFRSF1B
KLRC3
KLRC2
KLRF1
GZMK
Log2 fold change
F
LOAD exp vs sup
NK cells
S100B
Log2 fold change
G
S1PR5
UMAP_1
UMAP_2

Supplementary Figure 5: NK cell cytotoxicity correlates with CD8 T cell clonal expansion. (**A**) Differentially expressed genes between ALS and control meningeal NK cells. (**B**) Differentially expressed genes between PD and control meningeal NK cells (*left*) and corresponding upregulated GO terms (*right*). (**C**) Differentially expressed genes between EOAD and control meningeal NK cells. (**D**) Differentially expressed genes among meningeal NK cells from ALS samples with Superexpanded versus Expanded TCR repertoires (*left*) and corresponding upregulated GO terms (*right*). (**E**) Differentially expressed genes among meningeal NK cells from PD samples with Superexpanded versus Expanded TCR repertoires. (**F**) Differentially expressed genes among meningeal NK cells from LOAD samples with Superexpanded versus Expanded TCR repertoires. (**G**) UMAP highlighting cells expressing S1PR5. Blue color intensity corresponds to the relative level of expression of the selected gene in a cell.

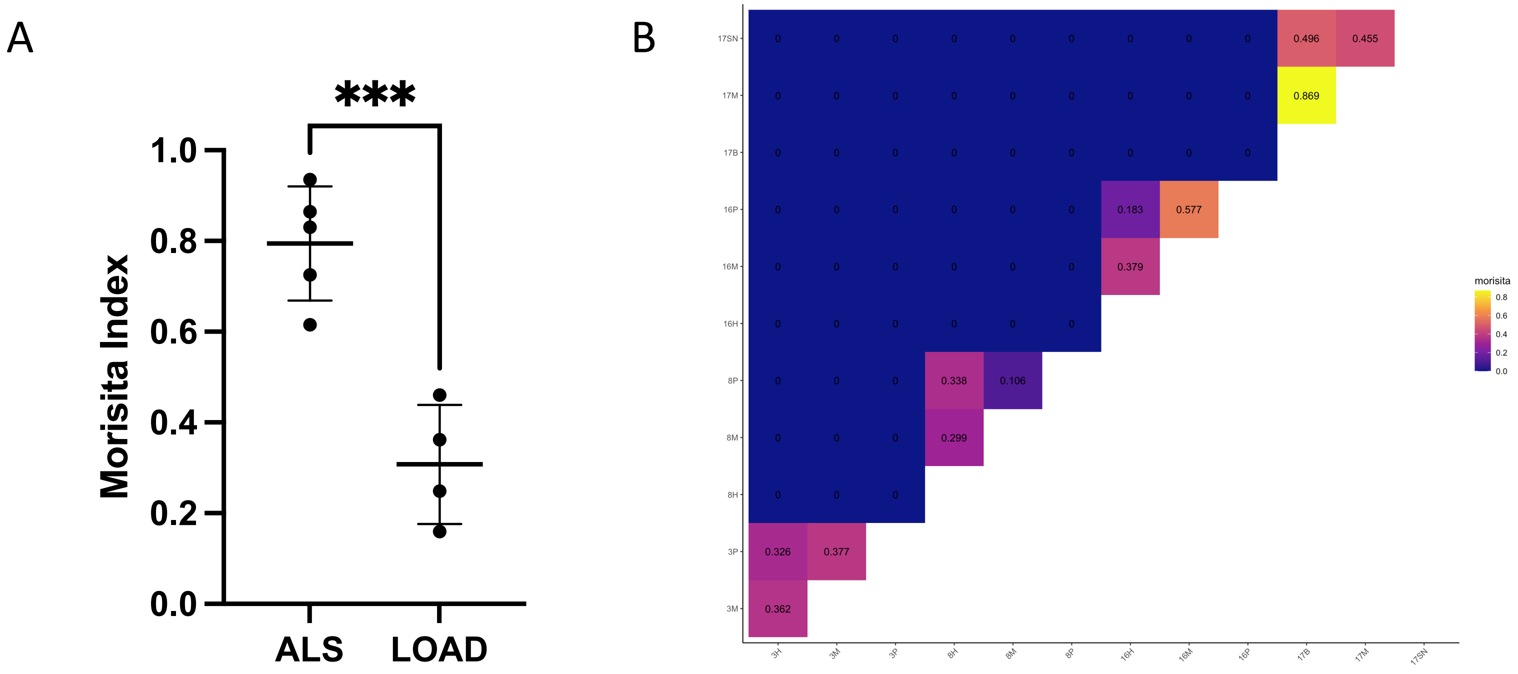

A
B
***
Morisita Index
1.0
0.8
0.6
0.4
0.2
0.0
ALS
LOAD
17SN
17M
17B
16P
16M
16H
8P
8M
8H
3P
3M
3H
0.496
0.455
0.869
0.183
0.577
0.379
0.338
0.106
0.299
0.326
0.377
0.362
morisita

Supplementary Figure 6: ALS diagnosis correlates with increased TCR sharing between cortex and meninges. (**A**) Morisita index between brain and meninges by disease. ALS and LOAD brain samples from motor cortex and hippocampus, respectively. (**B**) Morisita index measurement among meninges, hippocampus, and parietal lobe.

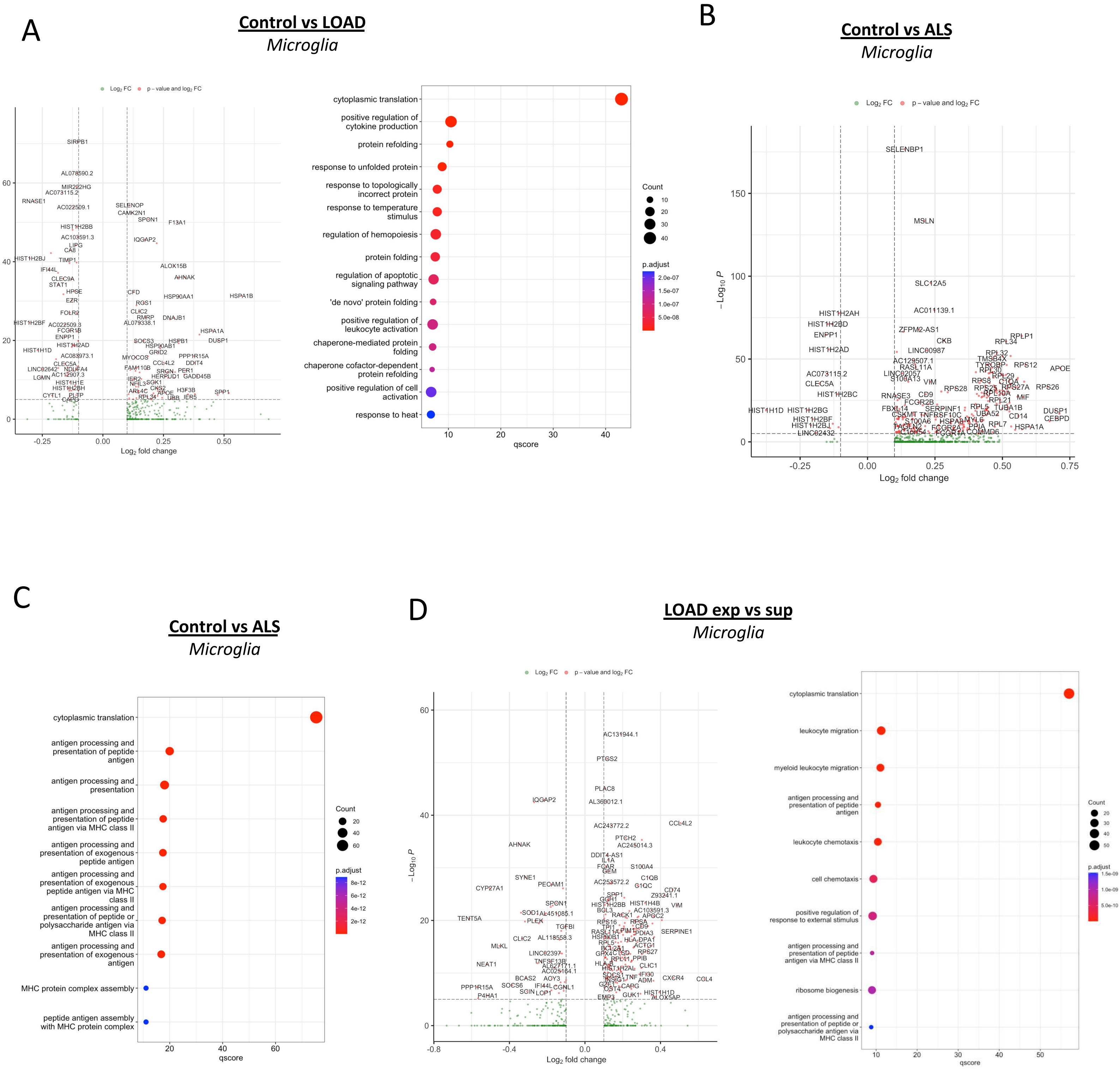
A
Control vs LOAD
Microglia
Log2 FC
p – value and log2 FC
−Log10 P
Log2 fold change
cytoplasmic translation
positive regulation of cytokine production
protein refolding
response to unfolded protein
response to topologically incorrect protein
response to temperature stimulus
regulation of hemopoiesis
protein folding
regulation of apoptotic signaling pathway
'de novo' protein folding
positive regulation of leukocyte activation
chaperone-mediated protein folding
chaperone cofactor-dependent protein refolding
positive regulation of cell activation
response to heat
qscore
Count
p.adjust
B
Control vs ALS
Microglia
Log2 FC
p – value and log2 FC
−Log10 P
Log2 fold change
SELENBP1
MSLN
SLC12A5
APOE
C
Control vs ALS
Microglia
cytoplasmic translation
antigen processing and presentation of peptide antigen
antigen processing and presentation
antigen processing and presentation of peptide antigen via MHC class II
antigen processing and presentation of exogenous peptide antigen
antigen processing and presentation of exogenous peptide antigen via MHC class II
antigen processing and presentation of peptide or polysaccharide antigen via MHC class II
antigen processing and presentation of exogenous antigen
MHC protein complex assembly
peptide antigen assembly with MHC protein complex
qscore
Count
p.adjust
D
LOAD exp vs sup
Microglia
Log2 FC
p – value and log2 FC
−Log10 P
Log2 fold change
AC131944.1
PTGS2
PLAC8
cytoplasmic translation
leukocyte migration
myeloid leukocyte migration
antigen processing and presentation of peptide antigen
leukocyte chemotaxis
cell chemotaxis
positive regulation of response to external stimulus
antigen processing and presentation of peptide antigen via MHC class II
ribosome biogenesis
antigen processing and presentation of peptide or polysaccharide antigen via MHC class II
qscore
Count
p.adjust

Supplementary Figure 7: Microglia contribute to meningeal T cell clonal expansion. (**A**) Differentially expressed genes between LOAD and control microglia (*left*) and corresponding upregulated GO terms (*right*). (**B**) Differentially expressed genes between ALS and control microglia. (**C**) GO terms inferred from genes upregulated in ALS microglia. (**D**) Differentially expressed genes among microglia from LOAD samples with Superexpanded versus Expanded TCR repertoires (*left*) and corresponding upregulated GO terms (*right*).

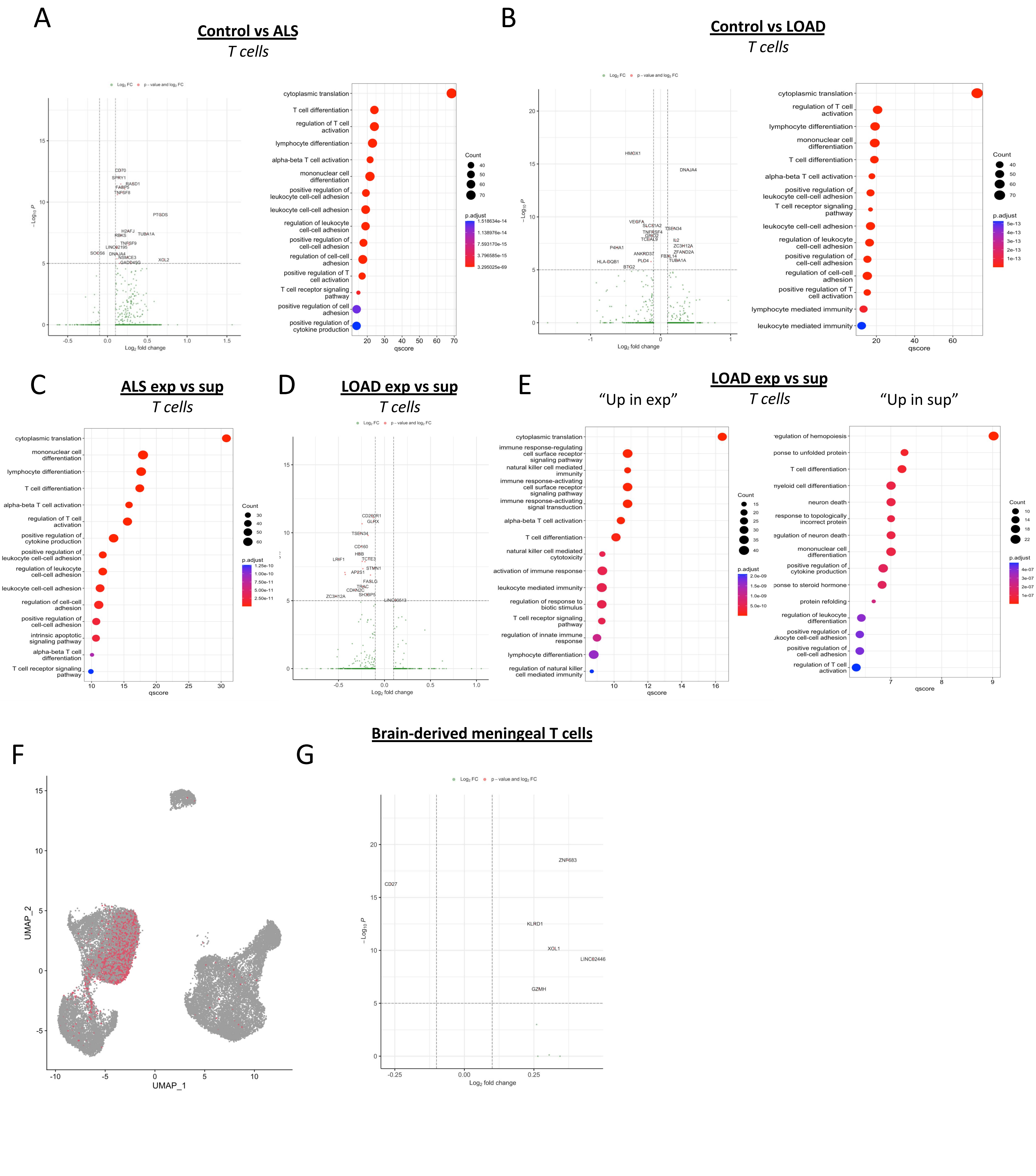
A
Control vs ALS
T cells
cytoplasmic translation
T cell differentiation
regulation of T cell activation
lymphocyte differentiation
alpha-beta T cell activation
mononuclear cell differentiation
positive regulation of leukocyte cell-cell adhesion
leukocyte cell-cell adhesion
regulation of leukocyte cell-cell adhesion
positive regulation of cell-cell adhesion
regulation of cell-cell adhesion
positive regulation of T cell activation
T cell receptor signaling pathway
positive regulation of cell adhesion
positive regulation of cytokine production
qscore
Count
p.adjust
Log2 fold change
B
Control vs LOAD
T cells
cytoplasmic translation
regulation of T cell activation
lymphocyte differentiation
mononuclear cell differentiation
T cell differentiation
alpha-beta T cell activation
positive regulation of leukocyte cell-cell adhesion
T cell receptor signaling pathway
leukocyte cell-cell adhesion
regulation of leukocyte cell-cell adhesion
positive regulation of cell-cell adhesion
regulation of cell-cell adhesion
positive regulation of T cell activation
lymphocyte mediated immunity
leukocyte mediated immunity
C
ALS exp vs sup
T cells
cytoplasmic translation
mononuclear cell differentiation
lymphocyte differentiation
T cell differentiation
alpha-beta T cell activation
regulation of T cell activation
positive regulation of cytokine production
positive regulation of leukocyte cell-cell adhesion
regulation of leukocyte cell-cell adhesion
leukocyte cell-cell adhesion
regulation of cell-cell adhesion
positive regulation of cell-cell adhesion
intrinsic apoptotic signaling pathway
alpha-beta T cell differentiation
T cell receptor signaling pathway
D
LOAD exp vs sup
T cells
E
LOAD exp vs sup
"Up in exp"
T cells
"Up in sup"
cytoplasmic translation
immune response-regulating cell surface receptor signaling pathway
natural killer cell mediated immunity
immune response-activating cell surface receptor signaling pathway
immune response-activating signal transduction
alpha-beta T cell activation
T cell differentiation
natural killer cell mediated cytotoxicity
activation of immune response
leukocyte mediated immunity
regulation of response to biotic stimulus
T cell receptor signaling pathway
regulation of innate immune response
lymphocyte differentiation
regulation of natural killer cell mediated immunity
T cell differentiation
neuron death
response to topologically incorrect protein
mononuclear cell differentiation
positive regulation of cytokine production
protein refolding
regulation of leukocyte differentiation
positive regulation of cell-cell adhesion
regulation of T cell activation
Brain-derived meningeal T cells
F
UMAP_1
UMAP_2
G
ZNF683
CD27
KLRD1
XCL1
LINC02446
GZMH
Log2 fold change

Supplementary Figure 8: T cells activated in the brain become meningeal resident T cells. (**A**) Differentially expressed genes between ALS and control brain T cells (*left*) and corresponding upregulated GO terms (*right*). (**B**) Differentially expressed genes between LOAD and control brain T cells (*left*) and corresponding upregulated GO terms (*right*). (**C**) GO terms inferred from elevated genes among brain T cells from ALS samples with Superexpanded versus Expanded TCR repertoires. (**D**) Differentially expressed genes among brain T cells from LOAD samples with Superexpanded versus Expanded TCR repertoires. (**E**) GO terms derived from genes upregulated in Expanded (*left*) or Superexpanded (*right*) LOAD brain T cells. (**F**) UMAP with meningeal cells expressing TCRs also found in the brain from the same patient highlighted in red. (**G**) Differentially expressed genes between meningeal resident T cells with a TCR also found in the brain versus all other meningeal resident T cells with a detected TCR sequence.

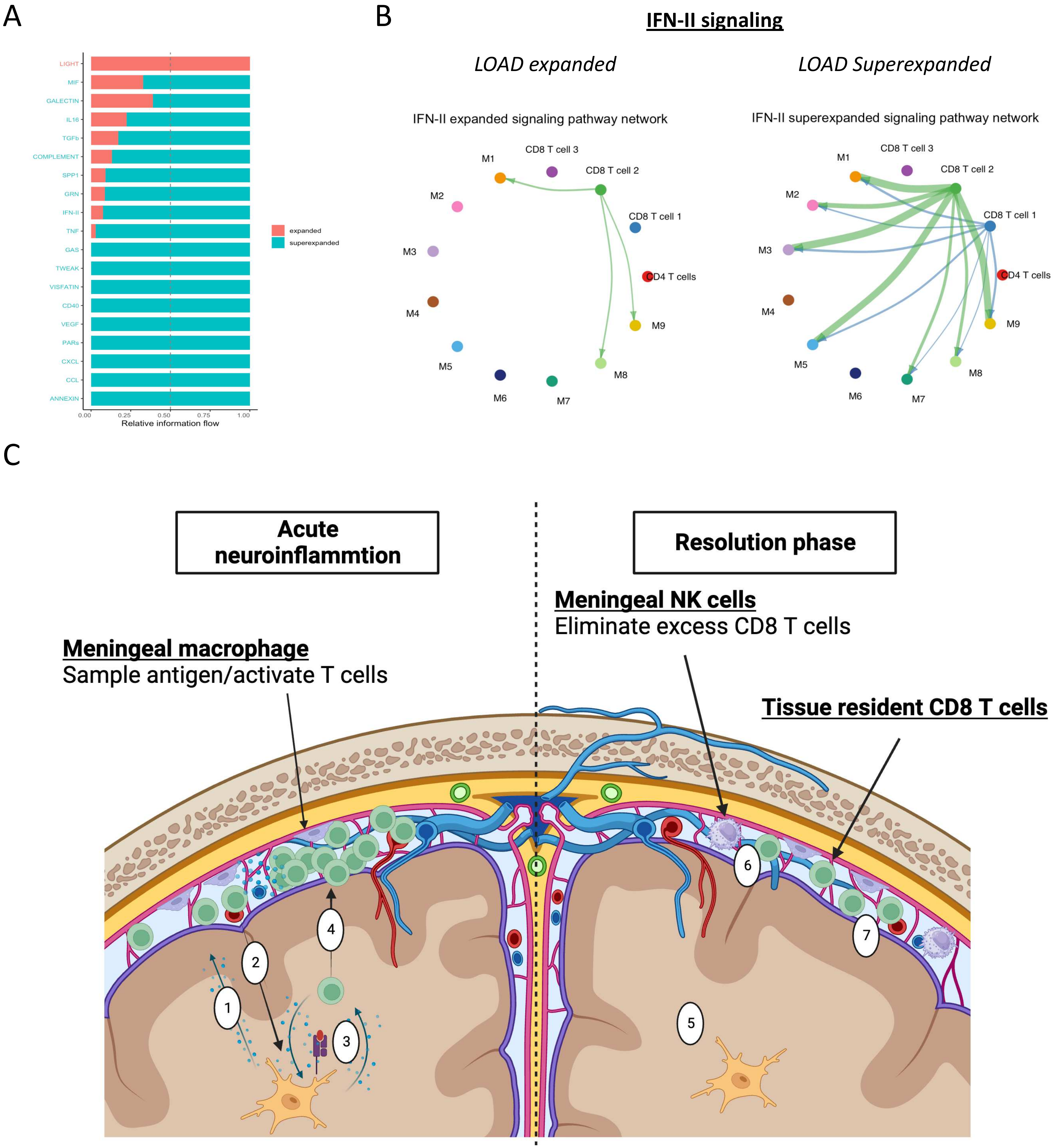
A
LIGHT
MIF
GALECTIN
IL16
TGFb
COMPLEMENT
SPP1
GRN
IFN-II
TNF
GAS
TWEAK
VISFATIN
CD40
VEGF
PARs
CXCL
CCL
ANNEXIN
0.00
0.25
0.50
0.75
1.00
Relative information flow
expanded
superexpanded
B
IFN-II signaling
LOAD expanded
LOAD Superexpanded
IFN-II expanded signaling pathway network
IFN-II superexpanded signaling pathway network
M1
CD8 T cell 3
CD8 T cell 2
M2
CD8 T cell 1
M3
CD4 T cells
M4
M9
M5
M8
M6
M7
C
Acute neuroinflammtion
Resolution phase
Meningeal NK cells
Eliminate excess CD8 T cells
Meningeal macrophage
Sample antigen/activate T cells
Tissue resident CD8 T cells
1
2
3
4
5
6
7

Supplementary Figure 9: Meningeal T cells interact remotely with the CNS. (**A**) Secreted intercellular ligand/receptor interactions inferred between meningeal T cells and microglia from LOAD patients using CellChat. Intercellular signaling pattern names highlighted in red on the y-axis denote interactions that were inferred as significantly upregulated in Expanded samples, and pattern names highlighted in blue represent interactions that were inferred as significantly upregulated in Superexpanded samples. (**B**) String diagrams depicting differential intercellular IFN-II signaling between meningeal T cells and microglia between Expanded and Superexpanded LOAD sample sets. Edge width represents the relative interaction strength detected between two cell clusters. (**C**) Graphic summary of proposed intercellular dynamics between meningeal and CNS immune cells. (*1*) Microglia secrete T cell homing cytokines which are detected by T cells in the meninges. (*2*) T cells migrate from the meninges into the brain where they are (*3*) activated by and contribute to microglial inflammation. (*4*) T cells egress to the meninges after performing their effector functions. (*5*) Microglia downregulate MHC and T cell homing cytokines and (*6*) meningeal NK cells upregulate killing programs to eliminate excess meningeal CD8 T cells. (*7*) Remaining meningeal CD8 T cells persist as tissue resident memory cells to be engaged in future insults.

Supplementary Files

This is a list of supplementary files associated with this preprint. Click to download.

- SupplementaryFile1.xlsx
- SupplementaryFile2.xlsx
